# Raw biomass electroreforming coupled to green hydrogen generation

**DOI:** 10.1038/s41467-021-22250-9

**Published:** 2021-03-31

**Authors:** Hu Zhao, Dan Lu, Jiarui Wang, Wenguang Tu, Dan Wu, See Wee Koh, Pingqi Gao, Zhichuan J. Xu, Sili Deng, Yan Zhou, Bo You, Hong Li

**Affiliations:** 1 School of Mechanical and Aerospace Engineering, Nanyang Technological University, Singapore, Singapore; 2 Advanced Environmental Biotechnology Centre, Nanyang Environment and Water Research Institute, Nanyang Technological University, Singapore, Singapore; 3 School of Materials Science and Engineering, Nanyang Technological University, Singapore, Singapore; 4 School of Materials, Sun Yat-sen University, Guangzhou, China; 5 Department of Mechanical Engineering, Massachusetts Institute of Technology, Cambridge, MA USA; 6 School of Civil and Environmental Engineering, Nanyang Technological University, Singapore, Singapore; 7 School of Chemistry and Chemical Engineering, Huazhong University of Science and Technology, Wuhan, Hubei China; 8 Centre for Micro-/Nano-electronics (NOVITAS), School of Electrical and Electronic Engineering, Nanyang Technological University, Singapore, Singapore; 9 CINTRA CNRS/NTU/THALES, UMI 3288, Research Techno Plaza, Singapore, Singapore

**Keywords:** Electrocatalysis, Sustainability, Chemical engineering

## Abstract

Despite the tremendous progress of coupling organic electrooxidation with hydrogen generation in a hybrid electrolysis, electroreforming of raw biomass coupled to green hydrogen generation has not been reported yet due to the rigid polymeric structures of raw biomass. Herein, we electrooxidize the most abundant natural amino biopolymer chitin to acetate with over 90% yield in hybrid electrolysis. The overall energy consumption of electrolysis can be reduced by 15% due to the thermodynamically and kinetically more favorable chitin oxidation over water oxidation. In obvious contrast to small organics as the anodic reactant, the abundance of chitin endows the new oxidation reaction excellent scalability. A solar-driven electroreforming of chitin and chitin-containing shrimp shell waste is coupled to safe green hydrogen production thanks to the liquid anodic product and suppression of oxygen evolution. Our work thus demonstrates a scalable and safe process for resource upcycling and green hydrogen production for a sustainable energy future.

## Abstract (web summary)

The scale-up of the coupling of water electroreduction (HER) with organic electrooxidation remains challenging. Here the authors address this challenge by coupling HER with electrooxidation of raw biomass chitin, cogenerating acetate and green hydrogen safely at high current density.

## Introduction

A sustainable development for the future needs conversion and storage of renewable energy that replenishes naturally and swiftly such as solar and wind. There are many challenges to integrate intermittent and unpredictable renewable energies to existing electric grids without causing interruption^1^. Extensive research has thus been devoted to energy storage devices such as battery systems, which could electrify the end-user sectors such as light-duty transport industry and residential buildings^2,3^. However, most of heavy-duty transportation including aircrafts and ships, and energy-intensive industries are difficult to be powered by electricity^4^. In addition, large-scale implementation of energy storage using batteries could be hampered by the low earth abundance of key materials including lithium and cobalt^5^. To this end, the storage of renewable energy in chemical energy carries is attractive to complement the electrical storage devices. Hydrogen fuel has been considered as one of the most promising chemical energy carriers due to its high energy density, free of greenhouse gas and pollutants emission, etc.^6,7^. Therefore, to store renewable energy in hydrogen-hydrogen chemical bonds in hydrogen molecules could hold the key to a truly sustainable energy future. Indeed, worldwide efforts have been devoted towards energy storage in hydrogen fuel such as power to gas^8^, power to X^9^, and H_2_@Scale initiatives (www.energy.gov/eere/fuelcells/h2scale), where hydrogen is generated by water electrolysis driven by renewable energies.

Water electrolysis is an industrial process for renewable and scalable green hydrogen generation. Among the three main water electrolysis technologies, namely alkaline water electrolysis (AWE)^10^, proton-exchange-membrane water electrolysis^11^, and solid-oxide water electrolysis^12^, AWE offers the most cost-effective and scalable method for renewable hydrogen generation^13^. Indeed, AWE contributes 2–3 million metric tons of hydrogen annually, i.e., about 4% global hydrogen production per year. The major obstacle for wider implementation of current water electrolysis technology is its much higher price (>$10 kg^−1^) than the dominant methane reforming technology (about $2 kg^−1^) (https://www.hydrogen.energy.gov/h2a_delivery.html)^14^. This could be mitigated by decreasing the electricity price that represents about 50% of the cost of AWE hydrogen production^15^. Thus, intensive effort has been devoted to the development of efficient catalysts^16^ or smart device architectures^17^ to lower electricity consumption. However, up to 90% of the electricity is consumed by the oxygen evolution reaction (OER) that couples to the hydrogen evolution reaction (HER)^18^. This thermodynamic constrain suggests significant reduction of electricity consumption is not possible in HER-OER couple.

Naturally, one may think of driving AWE using renewable energies such as solar or wind (could produce electricity at a price < $0.03 kWh^−1^)^19^. Nevertheless, the intermittent and uncontrollable solar or wind energy^20^ is not compatible with AWE due to partial load issue^21^, where serious gas crossover occurs when only partial electrolysis capacity (10–40% typically for AWE) is utilized. A gas mixture of hydrogen and oxygen is explosive at the working condition of electrolyzers^6^. As a result, AWE system has to be shut down to remove leaked H_2_ when its concentration reaches 2% in the oxygen compartment^22^. In order to completely avoid this partial load issue, separating HER from OER in time and space by a recyclable redox mediator has been investigated, including silicotungstic acid^23,24^, nickel hydroxide^25^, (ferrocenylmethyl)trimethylammonium chloride^26^, and anthraquinone-2,7-disulfonic acid^27^. Alternatively, one could replace OER with other reactions (e.g., organic oxidation reactions) that are thermodynamically more favorable, and produce liquid products on anode^28–31^. These strategies could successfully remove partial load issues and thus allow water electrolysis to couple to renewable energies directly. However, the scalability of hydrogen production is limited because none of these reactants (recyclable redox or organic oxidation reactions) could be as abundant as water, the reactant of HER and OER. In order to address this scalability mismatch issue, it is necessary to couple HER to an anodic reaction that oxidizes abundant reactants. As the most abundant natural amino biopolymer, chitin is ubiquitously found in insect skeleton, fungi, and crustacean shells with an annual production of 100 billion tons in nature^32,33^. A knowledge gap for raw biomass electroreforming arises from the rigid polymeric structure of raw biomass. Albeit chitin has comparatively low solubility in water, recent progress shows that optimized mechanochemical amorphization can greatly circumvent this barrier^34,35^.

In this study, we couple HER to the electrooxidation of the chitin^32^ for cogeneration of hydrogen and acetate, which could be further upgraded to a more valuable single-cell protein. We find chitin oxidation reaction (COR) is more favorable than OER thermodynamically and kinetically in the AWE setup. We have achieved a nearly complete chitin conversion to acetic acid (HAc)^36^. Such HER-COR couple offers a few apparent advantages. First, the hybrid electrolysis has excellent scalability with abundant reactants on both cathode (water) and anode (chitin), and higher efficiency can be obtained due to the more favorable reaction of COR compared to OER in traditional AWE. Second, the new redox couple completely removes the partial load issue since the by-product generated on the anode is dissolved in a liquid electrolyte, making AWE compatible with intermittent renewable energy. This feature allows membrane-free operation, making a single-compartment electrolyzer possible. Third, a much more valuable product (organic acids that can be further upgraded to protein) is produced on the anode, which can partially recover the operational cost. Since the product on the anode is almost pure acetate, no complicated separation, and extraction processes are required^37^. Lastly, COR offers significant environmental benefits since chitin is a major composition of solid waste^33,38,39^. Therefore, the bifunctional electrolysis for large-scale cogeneration of green hydrogen and commodity chemicals is highly beneficial for a sustainable future.

## Result and discussion

### Water electroreduction coupled with chitin electrooxidation

**Fig. 1:**
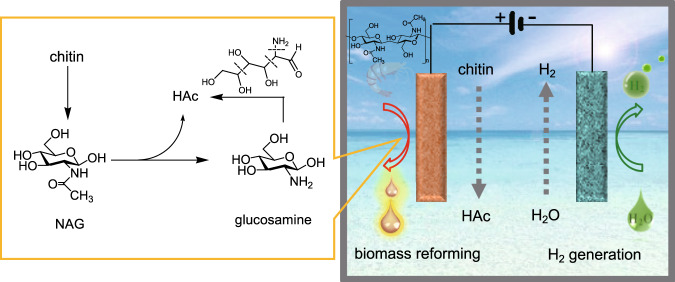
Hybrid electrolysis for raw biomass reforming and hydrogen evolution. Abundant biopolymer chitin exists in exoskeleton of insects and crustaceans. Electrochemical reforming of chitin selectively to acetic acid (HAc). Left panel: possible main reaction pathways. Right panel: schematic configuration of the hybrid electrolysis.

Figure 1 shows the schematic of the hybrid electrolysis system, where potassium hydroxide (KOH) solution serves as the electrolyte. HER occurs on the cathode to produce hydrogen (${\mathrm{H}}_2\mathrm{O} + e^ - \to \frac{1}{2}\mathrm{H}_2 + \mathrm{OH}^ -$), and COR proceeds on the anode to produce acetate. A membrane can be used to allow transport of OH^−^ ions between the cathode and anode while preventing oxidation products from reaching the cathode. A reaction pathway that produces main product of interest here is illustrated in the box (left). Depolymerization of chitin produces its monomer N-acetylglucosamine (NAG), which could be oxidized to produce HAc and glucosamine by deacetylation^37^. The opening of the pyranose ring in glucosamine will produce more HAc^37^. Since the products on the anode are soluble in the electrolyte, there is negligible gaseous product produced if COR dominates the anodic reactions. Moreover, the main product HAc is stable in the redox potential window (−0.4 to 1.7 V vs. RHE), and thus will neither be further oxidized on the anode nor be reduced on the cathode. As such, a membrane-free reactor can be employed for hybrid electrolysis. In addition, the hybrid electrolysis system can directly couple to renewable energy sources since the partial load issue is removed, as will be detailed later.

### Preparation of the cathode and anode

Three-dimensional hierarchical porous nickel (*hp*-Ni) catalyst was prepared by a facile template-free cathodic electrodeposition of porous Ni microspheres onto Ni foam (NF) substrate, i.e., *hp*-Ni/NF (see Supplementary Fig. 1). Hydrogen bubbles evolve at cathodic potential via HER during electrodeposition, responsible for the porous structure^30^. The anode was obtained by activating the as-prepared *hp*-Ni/NF electrode at a constant current density of 50 mA cm^−2^ until a stable OER performance was reached. During activation, the *hp*-Ni/NF electrode was used as the working electrode, and a Pt wire acted as the counter electrode. An Ag/AgCl (sat. KCl) electrode served as the reference electrode, which was calibrated with potassium ferricyanide prior to each usage. To fabricate the cathode, the surface of *hp*-Ni/NF electrode was converted to Ni_2_P/NF in a chemical vapor deposition system (see Methods and Supplementary Fig. 2 for details).

**Fig. 2:**
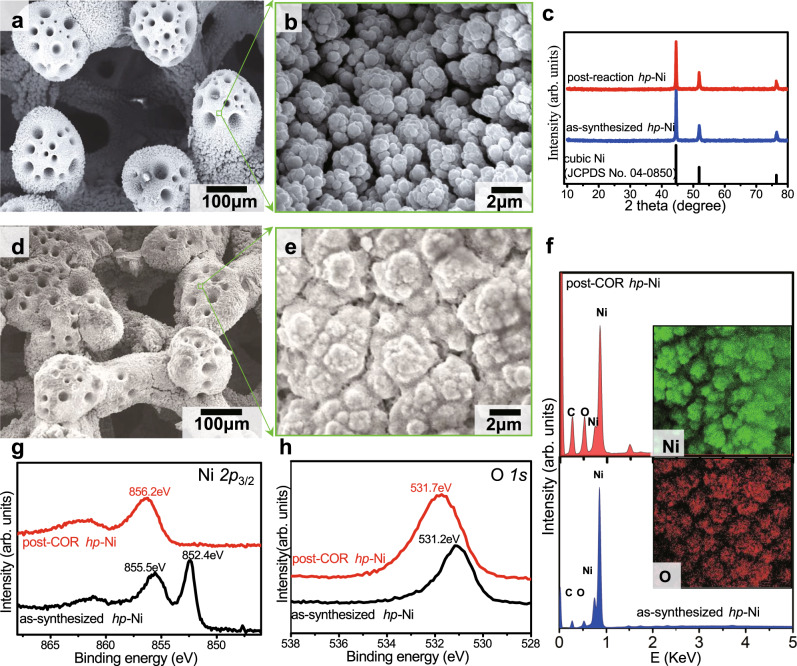
Material characterization of electrode. **a** SEM images of an as-synthesized *hp*-Ni sample. **b** Zoom-in view of the green square in (**a**). **c** X-ray diffraction patterns of as-synthesized (blue spectrum) and post-COR samples (red spectrum) along with the standard reference of cubic Ni (black spectrum). **d** SEM images of a post-COR *hp*-Ni sample. **e** Zoom-in view of the green square in (**d**). **f** EDS spectrum of as-synthesized (blue spectrum) and post-COR sample (red spectrum). Inset: Ni, and O element mapping of the post-COR sample. XPS spectrum of (**g**), Ni *2p* states, and **h** O *1s* state of the samples before (black spectra) and after (red spectra) COR.

Figure 2a depicts the scanning electron microscopy (SEM) images of the as-prepared *hp*-Ni/NF sample, which shows a clear 3D hierarchically porous features. The starting NF support was coated with a porous electrodeposited Ni film decorated with porous microspheres (100–200 μm in diameter). The NF skeleton has many pores with sizes ranging from micrometers to millimeters, and the porous microspheres also have numerous pores with sizes ranging from a few to tens of micrometers. The enlarged view of the green square area in Fig. 2a is displayed in Fig. 2b, which shows that the electrodeposited Ni film is composed of loosely packed Ni nanoparticles separated by nanoscale pores. As a result, the *hp*-Ni/NF electrode has a well-defined hierarchically porous structure with pores size ranging from nanometers to millimeters (see Supplementary Figs. 3 and 4 for more details). Such an electrode structure greatly facilitates mass transport and gas diffusion^30^, as well as exposes as many active sites as possible, which is responsible for the large current density observed. X-ray diffraction (XRD) pattern [the middle blue curve (as-synthesized *hp*-Ni) in Fig. 2c] shows that the *hp*-Ni/NF electrode contains crystalline cubic Ni (JCPDS card No. 04-0850), and this monolithic structure can endow the *hp*-Ni/NF electrode with high stability and excellent electrical conductivity. Such cubic Ni phase remained after the COR test [the upper red curve (post-reaction *hp*-Ni) in Fig. 2c]. The SEM images of the anode after the four-hour COR test (Fig. 2d, e) show that the integrity of the electrode structure was well retained, except for some tiny particles coating on the electrode surface, which could be the undissolved chitin crystals. The energy-dispersive X-ray spectroscopy (EDS) spectra (Fig. 2f) show that the oxygen element increases in concentration relative to that of the Ni element, owing to the anode activation and COR process. The elemental mappings (insets of Fig. 2f) further confirm that only nickel oxide/oxyhydroxide is present on the post-COR anode surface. Compared to that of the as-synthesized *hp*-Ni/NF, the peak at 852.4 eV on Ni *2p*_3/2_ X-ray photoelectron spectroscopy (XPS) spectrum that is ascribed to metallic Ni almost disappeared after COR (Fig. 2g, see Supplementary Fig. 5 for the full range spectra). This indicates the completed oxidation of the electrode surface, which is consistent with the increased peak at 856.2 eV. The much stronger peak on O *1s* XPS spectrum of post-COR anode further confirms that the COR process caused deeper oxidation of Ni surface (Fig. 2h). The enhanced nickel oxidation is very beneficial for high COR activity because the catalytic activity centers in Ni anode are the high-valent nickel species^40^.

### Electrochemical characterizations

**Fig. 3:**
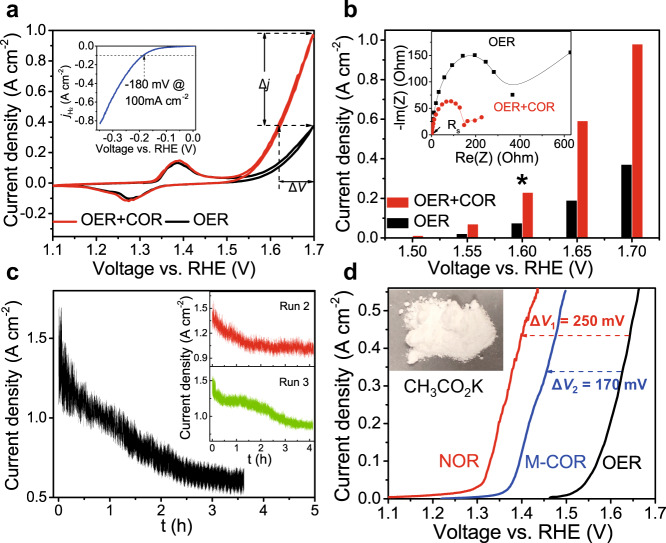
Electrochemical characterization. **a** Cyclic voltammograms of anodic reactions at scan rate of 10 mV s^−1^ in 1.0 M KOH before (OER; black curves) and after adding 33.3 mg L^−1^ of chitin (OER + COR; red curves). Δ*j* labels the current density difference at 1.7 V vs. RHE, and Δ*V* labels the potential difference at 0.4 A cm^−2^. Inset: Linear sweep voltammograms of HER using Ni_2_P/NF catalyst, where the potential for 100 mA cm^−2^ is labeled. **b** Comparison of current density at various potential in 1.0 M KOH solution with (red bars) and without (black bars) 33.3 mg L^−1^ chitin. Inset: the corresponding electrochemical impedance spectra (EIS) at open-circuit potential. *labels the voltage where the faradaic efficiency of oxygen evolution is characterized in Supplementary Fig. 10. **c** Consecutive three runs (black, red, and green curves represent the first, second, and third run, respectively) of chitin oxidation reaction (COR) in 1.0 M KOH with 33.3 mg L^−1^ chitin at a constant potential of 1.7 V vs. RHE. Inset: chronoamperometry curves of the second (upper) and third (lower) run. **d** Linear sweep voltammograms of anodic reactions at 5 mV s^−1^ in 1.0 M KOH (OER; black curve) and at the condition of containing 2.2 g L^−1^ N-acetyl glucosamine (red curve labeled with NOR) and 3.5 g L^−1^ milled chitin (blue curve labeled with M-COR). Arrow indicates the shift of potential after adding the corresponding biomass. Inset: a picture of 436 mg of oxidation product potassium acetate (CH_3_CO_2_K).

The anode was used to oxidize chitin with Ag/AgCl reference electrode and a Pt wire (or Ni_2_P/NF electrode) as counter electrode. A certain amount of chitin was dissolved in 1.0 M KOH solution by ultrasonic treatment for 30 min followed by a sequential freezing-thawing process (see Methods for more details)^41^. The scan rate was 10 mV s^−1^ in all cyclic voltammetry (CV) measurements. 85% *iR* compensation was applied to all current-voltage measurements to account for the Ohmic loss, where *R* was obtained by electrochemical impedance spectroscopy (EIS). The Ni_2_P/NF cathode is very active for HER in 1.0 M KOH electrolyte with a potential of −180 mV vs. RHE for current density of 100 mA cm^−2^, as displayed in the inset of Fig. 3a.

Figure 3a depicts the CV curves of the anode in 1.0 M KOH with (red) and without (black) 33.3 mg L^−1^ chitin, respectively. In the absence of chitin, the anode shows good OER activity in basic media with onset potential of 1.55 V vs. RHE. Significant OER current appears after Ni oxidation peak (Ni^2+^→Ni^3+^) at around 1.40 V vs. RHE, indicating Ni^3+^ is the active center for water oxidation^40^. In the presence of chitin, the anodic current emerges earlier and increases faster than the pure OER current. Specifically, the current density is Δ*j* ~ 600 mA cm^−2^ (or 2.5 times) higher at 1.7 V vs. RHE, and the potential is Δ*V*~ 80 mV lower at 0.4 A cm^−2^, than those of the pure OER curves, suggesting COR is kinetically more favorable than OER (see labels in Fig. 3a). It is noted that, at high overpotential, non-negligible OER could occur concurrently with COR, thus the observed current density comes from both OER and COR (OER + COR). COR and OER are believed to share the same active sites because the generated oxygen decreased when chitin was added, as demonstrated in Supplementary Fig. 6. The EIS spectra at open-circuit voltage (OCV) show that the series resistances *R*_s_ (left intersection point with *x*-axis) for both OER + COR (red circles) and pure OER (black squares) curves are similar (Supplementary Fig. 7 and the inset of Fig. 3b). In contrast, the charge-transfer resistance *R*_ct_ (diameter of the semicircle) of the OER + COR curve is 2.4 times lower than that of the pure OER curve (Supplementary Fig. 8 and Table 1). This comparison suggests that the much higher current density observed in CV scan (Fig. 3a) is caused by a much faster kinetics/reaction rate of COR than that of OER. Similar to OER, the COR current increases after the Ni oxidation peak, suggesting that high-valent Ni species are responsible for chitin oxidation. The differences between the current densities of OER + COR and OER at the same potential are summarized in Fig. 3b. One can see that the COR current (i.e., the difference between red and black bar) is larger than pure OER current in the full range of the potential applied, and selective chitin oxidation (negligible OER) is possible with a potential lower than 1.55 V vs. RHE. The generated oxygen was analyzed by gas chromatography with a thermal conductivity detector (GC-TCD), as displayed in Supplementary Fig. 9. It is confirmed that the generated oxygen is much less than the theoretical value (i.e., O_2_: H_2_ = 1:2). Faradaic efficiency of O_2_ at 1.6 V vs. RHE is about 30% (Supplementary Fig. 10), consistent with the current density ratio measured in Fig. 3b (labeled as *). The Chronoamperometry curve in Fig. 3c shows that the total current (OER + COR) decreases with the reaction duration. 33.3 mg L^−1^ of the initial chitin can be mostly converted in 3.5 h at a potential of 1.7 V vs. RHE. The stability and reusability of the anode for chitin oxidation were also evaluated by repeating oxidation of fresh chitin samples using the same anode. As shown in the inset of Fig. 3c, the activity of the anode for chitin oxidation can be well-preserved in three consecutive runs, which can also be corroborated by similar EIS cures (Supplementary Fig. 11). To significantly increase the chitin solubility in KOH electrolyte, we also conducted a mechanochemical pretreatment process (see Supplementary Fig. 12 and Methods for details). Oxidation of 3.5 g L^−1^ milled chitin (M-COR curve in Fig. 3d) shows much higher current density with an overpotential reduction of 170 mV owing to the increased solubility (Supplementary Figs. 13 and 14), as well as the thermodynamic and kinetic favorability (see Supplementary Fig. 15), due to the greatly reduced molecular weight that will be detailed later. In order to see the potential of COR in energy saving, we tested the monomer of chitin, NAG, which can dissolve well in the electrolyte. As shown in Fig. 3d, the NAG oxidation reaction (NOR) current with 2.2 g L^−1^ of NAG as reactant leaps at around 1.24 V vs. RHE, which is much lower than the onset potential of OER around 1.49 V vs. RHE. As a result, highly selective NAG oxidation (without OER) occurs in the potential window ranging from 1.24 to 1.49 V vs. RHE. Such a low onset potential of NOR indicates that Ni^2+^ is able to oxidize NAG. The 250 mV reduction in OER overpotential corresponds to 15% energy saving in electrolysis. It is worth noting there is negligible oxygen detected by GC-TCD up to 1.45 V vs. RHE (Supplementary Fig. 16). Furthermore, a previously reported precipitation method was adopted to extract potassium acetate product (see Supplementary Fig. 17 and Methods for details). An optical image of the powder of potassium acetate hydrate (CH_3_CO_2_K·xH_2_O) product is inserted in Fig. 3d, with the detailed characterizations shown in Supplementary Figs. 18–20. It is noted that the energy ΔG needed for pyranose-ring opening and glycosidic bond breaking is much lower than that of water splitting^42–44^. This is consistent with the observed much lower onset potential of NOR than that of OER. However, the thermodynamic advantage of COR over OER is much smaller due to the high degree of polymerization of NAG in chitin, resulting in the extra energy barrier arising from depolymerization.

### COR product identification and quantification

**Fig. 4:**
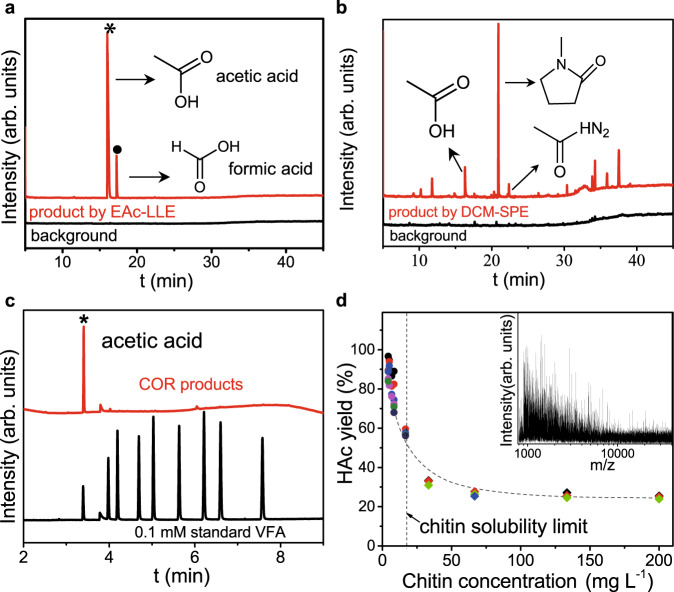
Identification and quantification of products. Gas chromatography-mass spectrum (GC-MS) analysis of anodic reaction products after **a**, liquid–liquid extraction (LLE) and **b** solid-phase extraction (SPE). The black curves in (**a**) and (**b**) are spectra of background. The corresponding chemicals associated with the main peaks are labeled. **c** GC-FID quantification of acetic acid. The spectrum of the standard VFAs sample (black spectrum) is shown for comparison. **d** Yield curve of acetic acid. The vertical dotted line labels the chitin solubility limit using freeze-thawing pretreatment. Inset: MALDI-TOF mass spectra of freeze-thawing dissolved chitin in 1.0 M KOH solution. Each data point represents an individual sample.

The oxidation products on anode were first identified by GC-mass spectrometry (GC-MS) method after liquid–liquid extraction (LLE) and solid-phase extraction (SPE) using acetyl acetate (EAc) and dichloromethane (DCM) as the solvent and SPE cartridge as the solid-phase column, respectively (see Methods for details). In order to test all possible COR products/ intermediates, we collected the products at various stages of reaction before the reactant was fully converted. LLE extraction using EAc solvent (red spectrum in Fig. 4a) shows volatile fatty acid (VFA) products, which contains dominantly HAc with minor formic acid, in obvious contrast to flat background spectrum (solvent only). SPE extraction result (Fig. 4b) shows N-containing chemicals, where NMP appears to be the dominant one. Various other intermediate products were also found by other extraction methods, as shown in Supplementary Fig. 21. To quantify the yield of HAc, GC with flame-ionization detector (GC-FID) measurement was performed, as shown in Fig. 4c. The spectrum of COR product after completely converting 133.3 mg L^−1^ of chitin, along with that of the control sample consists of 0.1 mM of standard VFAs, indicates a HAc concentration of 0.5 mM. In order to find out the solubility of chitin in our electrolyte, total carbon content (TOC) was measured and then plotted against added carbon (by weighing chitin reactant), as depicted in Supplementary Fig. 13. One can see that our freeze-thaw method (see Methods) is able to achieve chitin solubility of >= 8.3 mg L^−1^ in 1.0 M KOH solution. HAc yield from COR was then calculated by the ratio of carbon in HAc product to that in the chitin reactant. As shown in Fig. 4d and Supplementary Fig. 22, the completely dissolved chitin could be effectively oxidized to HAc with over 90% yield, corroborating the opening of pyranose rings in chitin. The polymeric nature of the freeze-thawing dissolved chitin was further confirmed by matrix-assisted laser desorption/ionization-time of flight-mass spectrometry (MALDI-TOF-MS) (inset of Fig. 4d) and ^1^H nuclear magnetic resonance (NMR) spectra (Supplementary Fig. 23). The possible reaction pathways are illustrated in Supplementary Fig. 24 based on the intermediates and products identified. First, chitin is depolymerized and deacetylated under anodic condition to afford glucosamine, releasing HAc from the side chain. Afterward, most glucosamine is further hydrolyzed to open the ring, followed by several oxidations and bond-breakings. All intermediates are unstable under the oxidative environment and finally converted into small organic acids as the reaction progresses. Due to the highly oxidative reaction conditions, only HAc and minor formic acid were collected eventually. On the other hand, minor glucosamine undergoes dehydration to form furanic compounds, followed by either oxygen-nitrogen exchange to produce N-methyl-2-pyrolidone, or rupture of amine group to afford tetrahydrofurfuryl alcohol that have all been detected.

### Single-compartment hybrid electrolyzer driven by solar energy

We accessed the feasibility of safe hydrogen production driven by intermittent solar energy via PV panels on the roof of our laboratory (N 1°20′54.4″; E 103°40′59.3″; 2–5 pm on 14 June 2019; partially cloudy). Commercial PV panels (maximum output power 50 W each) were employed to power a sealed single-compartment reaction cell (see Supplementary Fig. 25 and Movie 1 for the detailed setup), where the generated gaseous products were collected by using a few gas sampling bags (1 L each). Supplementary Fig. 26a shows the filling of sampling bags by gaseous products at four different reaction times (0, 12.5, 24, 51.3 min) with milled chitin as the reactant (see the inset of Supplementary Fig. 26b for the homemade sealed reactor). The oxygen concentrations of the gaseous products in the 4 sampling bags collected sequentially are depicted in Supplementary Fig. 26b, and the corresponding faradaic efficiencies of COR and OER at different reaction time are shown in Supplementary Fig. 27. One can see that the oxygen concentration of the gas products remains below the exposure limit of hydrogen-oxygen mixture (6% O_2_ in H_2_-O_2_ mixture as indicated by the dashed line) for the first 3 sampling bags despite the fluctuating output power from the PV panels (between 28.5 and 54 W) due to the presence of moving clouds. The recorded PV output voltage (from voltmeter) varied between 3.0 and 3.6 V, and the *iR*-corrected voltage range was 2–2.1 V (see Supplementary Table 2). It’s worth noting that more oxygen evolved when the reactant concentration decreased in the electrolyte as the reaction progressed. Supplementary Fig. 26c shows the current density response when fresh reactants (5 mL every time) were added four times sequentially to the reactor (25 mL milled chitin with a concentration of 3.5 g L^−1^). One can see an apparent current leap upon the addition of fresh reactant, suggesting M-COR dominated the reactions. When the reactant concentration remained high, the O_2_ concentration in the gaseous product remained low. Therefore, a fluidic cell with constantly added reactants should be employed to further minimize OER. Supplementary Fig. 26d shows the H_2_ gas production rate derived from the collected H_2_ gas, which reaches 0.073 L min^−1^ (electrode area of 28 cm^2^). It is noted that the catalyst remained active after repeated cycles of reaction (Fig. 3c and the inset) and prolonged reaction time (Supplementary Fig. 28), suggesting the anode is stable for both COR and OER. From the evolution of the reactant color with increased reaction time (Supplementary Fig. 29), one can see that the electrolyte rapidly became clearer in the first 3-h reaction, and then color slowly faded. Eventually, the electrolyte became almost clear after 24-h reaction. The final product consisted of mainly HAc with minor formic acid (<5%) as suggested by GC-MS (Supplementary Fig. 30), GC-FID (inset of Supplementary Fig. 26d), and high-performance liquid chromatography (HPLC) measurements (see Methods for details). The yield of HAc was obtained to range from 73.7 to 77.5% from batch to batch.

Our proof-of-concept single-compartment AWE prototype shows great compatibility with intermittent solar energy. With decreasing the price of PV and wind electricity to $0.02 kW h^−1^, conventional AWE can be more cost-effective ($ 100–400 kW^−1^) than methane reforming^15^. Our hybrid electrolysis will further reduce the price of green hydrogen production via water electrolysis. Moreover, direct coupling of renewable energy to electrolyzer avoids the integration of renewable energy to electrical grid, which faces many challenges currently^1^. This will also reduce electricity consumption in various AC/DC converters, voltage/current regulators, etc. Lastly, the single-compartment electrolyzer simplifies the overall device and system design, and thus reduces the cost of manufacturing, maintenance, and operation. Albeit the energy consumption of ball mill at current stage still hinders the practical application of our hybrid electrolysis to some extent, further reduction of energy consumption by using the appropriate cocatalyst or/and increasing the processing scale (see Supplementary Notes, Figs. 31 and 32, and Tables 4–7 for more details). Therefore, such a hybrid AWE will facilitate the wide implementation of water electrolysis by potentially reduced cost and increased safety, highly beneficial for a sustainable energy future.

### Electrochemical conversion of crude shrimp shells

**Fig. 5:**
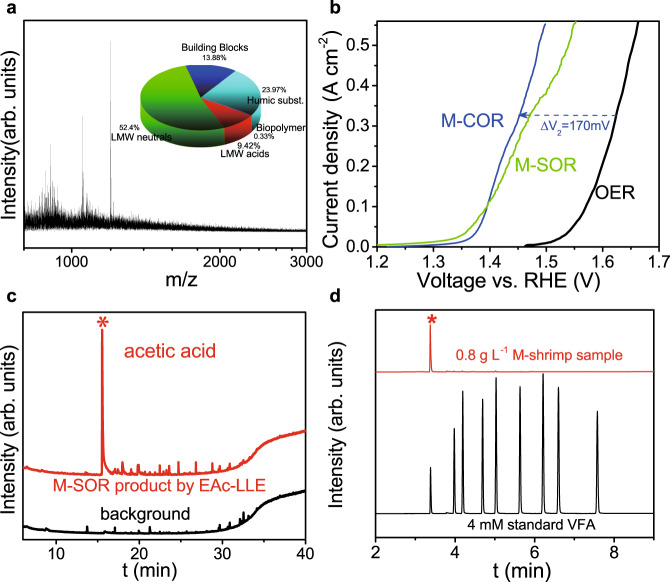
Electrochemical conversion of M-shrimp shell. **a** MALDI-ToF mass spectra of soluble products from ball-milled crude shrimp shell. Inset: soluble substances fractionation of ball-milled shrimp shell. **b** LSV of anodic reactions at 5 mV s^−1^ in 1.0 M KOH with 6.0 g L^−1^ milled shrimp shell (green curve labeled with M-SOR). The anodic OER (black curve) and M-chitin oxidation reaction (blue curve labeled with M-COR) curves are also plotted for comparison. **c** GC-MS identification of anodic oxidation product of ball-milled shrimp shell. The black spectrum represents the background. **d** GC-FID quantification of acetic acid. The spectrum of standard VFAs sample (black spectrum) is shown for comparison.

To further extend the sustainability of our hybrid electrolysis, crude shrimp shell (as a replacement for chitin) from seafood waste was fed to the electrolyzer. Shrimp shells (Caridina heteropoda), which contain 29 wt% calcium carbonate (CaCO_3_), were pretreated under the same kaolinite-assisted ball milling process as chitin pretreatment, except that different ratio was used. CaCO_3_ remained insoluble after ball milling and was removed by a simple filtration after dispersing the products in water. While the rest ~71 wt% mass (mainly protein and chitin) underwent partial depolymerization and amorphization under the mechanochemical treatment and then dissolved in water. The MALDI-TOF-MS result (Fig. 5a) shows that the molecular weight of the dissolved section mainly locates in the range of 800–1300 Da, while the results from size exclusion chromatography coupled with organic carbon detection and organic nitrogen detection (SEC-OCD-OND) (see the inset of Fig. 5a) demonstrate that the main categories of the dissolved section are humic substance and low molecular weight polysaccharide. Oxidation of 6.0 g L^−1^ milled shrimp shell (M-SOR curve in Fig. 5b) shows similar current density increase as M-COR, and even lower onset potential. GC-MS characterizations (Fig. 5c) show that the main product is still HAc with trivial formic acid. And the GC-FID (Fig. 5d) quantification shows that the yield of acetic acid reached up to 42 toc%. It is worth noting that our electrolyzer shows significantly higher current density than the reported electrochemical conversion of lignocellulose^45–64^, suggesting a big step towards practical use (see detailed comparison in Supplementary Table S3).

In this work, we employed COR to replace OER in traditional AWE. Electrocatalytic oxidation of chitin that produces high-purity organic acids is coupled to water reduction. Such modified electrolysis is much safer than traditional water electrolysis because the product acetate is dissolved in the electrolyte, and thus avoids the formation of the explosive gas mixture with hydrogen. This feature allows direct coupling of intermittent solar energy to water electrolysis because the partial load issue is addressed. A solar energy-driven membrane-free single-compartment reactor is demonstrated to produce hydrogen gas at the rate of 73 mL per min with over 70% yield of acetic acid from chitin. Most importantly, chitin oxidation is as scalable as water reduction for hydrogen generation because chitin is produced more than 100 billion tons annually in nature. Chitin-containing waste shrimp shell also shows a high conversion rate, suggesting the excellent sustainability of the process. Putting together, the new hybrid electrolysis system has a few advantages over the traditional water electrolysis including (1) free of partial load issue and thus compatible with intermittent renewable energy sources, (2) electrooxidation of chitin reforms raw biomass to commodity chemicals in a green process, (3) much safer operation due to highly soluble products generated on anode, (4) much lower cost due to cheap renewable electricity, simple device configuration, and easy operation, (5) much more valuable product (organic acid) than oxygen is produced on anode, and (6) significant environmental benefit by upcycling chitin/shrimp shell waste, a major composition of solid waste. Therefore, our work paves the way towards a safe and scalable raw biomass electroreforming and green hydrogen production for a sustainable future.

## Methods

### Chemicals

Ammonium chloride, nickel sulfate hexahydrate, potassium hydroxide, potassium ferricyanide, formic acid, sodium sulfate, dichloromethane, ethyl acetate, kaolinite, and chitin were purchased from Sigma-Aldrich, hydrochloric acid and methanol were purchased from VWR, Sodium hypophosphite monohydrate was purchased from Alfa Aesar, Ag/AgCl (sat KCl) was purchased from CHI, and other chemicals were purchased from commercial vendors. All chemicals were used as received without further purification. Deionized water (18 MΩ cm) was used in all experiments.

### Synthesis of *hp*-Ni/NF

*hp*-Ni/NF anode was prepared by a facile template-free cathodic electrodeposition of porous Ni microsphere arrays on a pre-shaped Ni foam (0.5 × 0.5 cm^2^). Typically, the deposition was performed in a standard two-electrode configuration at room temperature with an electrolyte of 2.0 M NH_4_Cl and 0.1 M NiSO_4_. Pre-shaped commercial Ni foam and platinum wire were used as the working electrode and counter electrode, respectively. The electrodeposition was carried out at a constant current of −2 A cm^−2^ for 500 s to obtain *hp*-Ni/NF samples.

### Synthesis of Ni_2_P/NF

Ni_2_P/NF was synthesized by phosphorization of the as-synthesized *hp*-Ni/NF according to the literature with minor modification^65^. Specifically, the *hp*-Ni/NF was placed at the center of a tube furnace, with 1.0 g of NaH_2_PO_2_·H_2_O at the upstream near *hp*-Ni/NF. After flush with argon, the furnace was quickly heated up to 400 °C at a ramping rate of 10 °C min^−1^ and kept at 400 °C for 2 h. Then, the temperature of the furnace was naturally cooled down to room temperature.

### Pretreatment of chitin

The kaolinite-assisted milling experiments were conducted on ball mill Emax (Retsch) with two 125 mL zirconium oxide grinding jars and 0.5 mm size zirconium oxide grinding balls. In order to maximize the solubility of milled chitin samples, the milling conditions (packing degree, milling speed, and the ratio of chitin and kaolinite) were optimized. Similar to the former result, higher milling speed and packing degree resulted higher solubility^35^. Specifically, 3.0 g chitin and 6.0 g kaolinite were loaded into each grinding jar with 70% packing degree of grinding balls, and milled under 2000 rpm for 6 h. The temperature of the grinding jar was controlled by the machine built-in temperature controller not to exceed 65 °C during the whole milling process. The milled chitin samples were washed with a specific volume of DI water and centrifuged to recycle kaolinite. The precipitated kaolinite could be reused, while the dissolved chitin was collected as a reactant of hybrid electrolysis.

### Pretreatment of raw shrimp shell

A similar ball-mill procedure was applied to crude shrimp shell waste. The crude shrimp shells used were glass shrimps (Caridina heteropoda) shells^34^ bought from a local supermarket, which were peeled off (no flesh residue) directly from shrimps, followed by air dried and ground to powders with a blender. Prior to ball-mill decomposition, similar quantification method was employed to confirm the similar content of chitin, CaCO_3,_ and protein as that in the previous reports^34^. The kaolinite-assisted milling experiments were the same as the pretreatment of chitin, except 6.0 g crude shrimp shell and 6.0 g kaolinite were used as the feed in each jar.

### Material characterizations

SEM and element mapping were conducted on an FESEM 7600F. X-ray diffraction patterns were recorded on a Shimadzu XRD-6000. The X-ray photoelectron spectroscopy analyses were performed using a Kratos Axis Supra Spectrophotometer. X-ray photoelectron spectra were collected using the monochromatic Al Kα source (1486.7 eV) at a 300 × 700 μm^2^ spot size. Low-resolution survey and high-resolution region scans at the binding energy of interest were collected for each sample. To minimize charging, all samples were flooded with low-energy electrons and ions from the instrument’s built-in charge neutralizer. The samples were also sputter cleaned inside the analysis chamber with 1 keV Ar^+^ ions for 30 seconds to remove adventitious contaminants and surface oxides. All XPS data were fitted using Shirley background together with Gaussian-Lorentzian function using CASA XPS software, and energy corrections were calibrated by referencing the C *1s* peak of adventitious carbon to 284.8 eV.

### Electrochemical measurements

1.0 M KOH solution was chosen as the basic electrolyte, and different types of chitin-related biomass were added to study the corresponding oxidation process. First, a certain amount of chitin was dissolved in 1.0 M KOH solution by 0.5-h ultrasonic treatment and the sequential freezing process from room temperature to −20 °C. Before the electrooxidation process, the chitin solution was defrosted at room temperature. Ball milling pretreatment of chitin and raw shrimp shells were also adopted to replace freeze-thawing process. The electrooxidation process was performed on a Bio-logic vsp-300 electrochemical workstation with a three-electrode glass cell configuration at room temperature. The *hp*-Ni/NF was used as the working electrode, Pt wire or Ni_2_P/NF acted as the counter electrode, and Ag/AgCl (sat. KCl) electrode was employed as the reference electrode. The reference electrode was calibrated with potassium ferricyanide prior to each usage. All newly synthesized anode catalysts were activated at a constant current density of 50 mA cm^−2^ until a stable OER performance was obtained. After the electrochemical activation process, the *hp*-Ni/NF electrode was used as the anode to electrooxidize chitin or chitin-containing shrimp shell waste. Ten cycles of cyclic voltammetry (CV) scan was performed before each chronopotentiometry transformation of chitin. All scan rates were set at 10 mV s^−1^ unless specified. All the potentials reported were converted from vs. Ag/AgCl to vs. RHE (reversible hydrogen electrode) by adding a value of 0.197 + 0.059*pH. The 85% *iR* compensation was applied with R obtained from EIS measurement to account for the parasitic Ohmic loss.

### Purification of synthesized acetate and cycling of the system

A similar purification method as previous report^66^ was used with minor modification. Liquid post-reaction solution was concentrated at 40 °C under vacuum to viscous state. This concentrated product was further cooled to 0 °C in an ice bath. A fleck of potassium acetate was added to initialize crystallization. The product was allowed to crystallize at 0 °C for 30 min. The crystalline slurry was then filtered over a medium-porosity glass frit and compacted to aid the removal of any excess KOH solution from the crystalline product. The resulting solid was further dried at 80 °C under vacuum condition for one day to get acetate hydrate product with purity of 99%. The remaining KOH solution can be further recycled for the next run of electrolysis, as depicted in Supplementary Fig. 33.

### ^1^H NMR and MALDI-TOF-MS analysis

MALDI-TOF-MS mass spectra were obtained using a Bruker Autoflex III Smartbeam TOF/TOF 200 system equipped with a nitrogen laser (337 nm, 200 Hz maximum firing rate) with a mass range of <400,000 Da. The sample was prepared by mixing 1 μL product solution with 1 μL saturated 2,5-dihydroxybenzoic acid (2,5-DHB) matrix solution. ^1^H NMR spectra were recorded on Bruker AVANCE spectrometers operating at 500 MHz at room temperature. Prior to MALDI-TOF-MS and NMR testing, the freeze-thawing dissolved chitin sample was filtered through 4.5 μm membrane. In order to obtain qualitative molecular weight information of freeze-thawing dissolved chitin, commercial chitosan dissolved in diluted HCl solution and N-acetyl glucosamine in DI water was used as reference under the same testing condition. For the NMR quantification of the collected crystal solid, a certain concentration of collected solid in deuterated water was prepared and maleic acid was used as internal standard. And the purity of acetate was calculated by the ratio of the tested concentration of acetate to the prepared concentration of acetate.

### Quantification of gaseous product

All reactions were performed in a single-compartment electrochemical cell with *hp*-Ni/NF, Pt wire, and Ag/AgCl (sat. KCl) as the working, counter, and reference electrode, respectively. Before each reaction, we purged argon for 20 min to remove the air in the cell. After specific reaction time or passing a specific amount of charge, 0.5 mL gas was injected into GC-TCD (Agilent 6890 N, TCD detector, argon as carrier gas, 5 Å molecular sieve column) for quantification. For OER + COR reactions, the electrolyte was 1.0 M KOH solution with 33.3 mg L^−1^ of chitin. The potential was held constant at 1.6 V vs. RHE. For NOR reaction, the electrolyte was 1.0 M KOH solution with 2.2 g L^−1^ of NAG, and the potential was varied from 1.25 V to 1.5 V vs. RHE. For oxidation of milled chitin (M-COR), the electrolyte was 1.0 M KOH solution with 3.5 g L^−1^ M-chitin, and the applied potential range was 1.35–1.5 V vs. RHE. While for the solar energy-driven two-electrode cell, the electrolyte was 1.0 M KOH solution with 5.0 g L^−1^ dissolved M-chitin.

### Biomass oxidation product identification

The identification of products and intermediates were performed on GC-MS (gas chromatography-mass spectrometry, GCMS-QP2010ULTRA, Shimadzu) with ZB- WAXplus (Phenomenex) column of 30 m × 0.25 mm with a film thickness of 0.25 μm. For the intermediate’s identification, the reaction solutions at different reaction time, ranging from 10 min to 2 h, were collected. For product identification, 4 runs of reaction solution were collected for further extraction. Prior to extraction, the reaction solutions were acidified with 37 wt% HCl solution to pH value of 2.0. The Acidified solutions were further liquid–liquid extracted using dichloromethane (DCM) and ethyl acetate and solid–liquid extracted with cartridges (Waters Oasis®HLB, Waters Corporation, United States), respectively. Three microliters of extracted samples were injected for each test. The splitless injection was used at 240 °C with helium as a carrier gas at 1 mL min^−1^. The temperature program was as follows: 50 °C, hold 7 min, increase 7 °C min^−1^ to 240 °C, hold for 14 min. The MS was operated in electron ionization (EI) mode with the ion source temperature at 220 °C, and mass spectra were acquired from *m/z* 30 to 500 after a 5-min solvent cut time. The peaks were identified using the NIST11 library (National Institute of Standards and Technology, Gaithersburg, MD, http://www.nist.gov/srd/mslist.htm), and a compound was identified if the match was >80% (most matches were considerably higher).

### Biomass oxidation product quantification

The total organic carbon (TOC), inorganic carbon (IC), and total nitrogen (TN) in reaction liquid was analyzed by using TOC/ TN analyzer (Shimazu, Japan). The HAc product was quantified by GC-FID (GC7890A, Agilent, USA) with DB-FFAP fused – silica capillary column. Prior to product quantification, the reaction solution was neutralized with 37% HCl solution to pH value of 6.4. For GC testing, 900 μL neutralized reaction liquid was further acidified by mixing with 100 μL of formic acid. Agilent 1260 HPLC with 7.7 mm × 300 mm PL Hi-Plex H column and VWD (210 nm) detector was used to further quantify organic acid product. The mobile phase was 5 mM H_2_SO_4_ with flow rate of 0.6 mL min^−1^, and the detection temperature was 25 °C.

Yield (%) = mass of carbon in product/ mass of carbon in (chitin/ shrimp shell) reactant × 100%.

### Solar energy-driven single-compartment AWE cell

A homemade sealed single-compartment cell was connected to three commercial PV cells in parallel (power of 50 W each from Zibo Guosheng Solar Power Company) through a slide rheostat (variable resistance in the range of 0–1 Ω). Two pieces of enlarged *hp*-Ni/NF and Ni_2_P/NF with surface area of 28 cm^−2^ were used as anode and cathode respectively. The experiments were conducted on the roof of our laboratory (N 1°20′54.4″; E 103°40′59.3″) at 2–5 pm on 14 June 2019 (partially cloudy).

## Supplementary information

Supplementary Information

Description of Additional Supplementary Files

Supplementary Movie 1

## Data Availability

The data that support the findings of this study are available from the corresponding authors on request.
