# Supplementary material for: Raw biomass electroreforming coupled to green hydrogen generation
Source: Nat Commun. 2021 Mar 31;12:2008. doi: 10.1038/s41467-021-22250-9 (PMC8012647; doi:10.1038/s41467-021-22250-9)
Supplement: Supplementary file 1 — Supplementary Information [file 41467_2021_22250_MOESM1_ESM.pdf]

Supplementary Information

Raw Biomass Electroreforming Coupled to Green Hydrogen Generation

Hu Zhao[1†], Dan Lu[2†], Jiarui Wang[3], Wenguang Tu[1], Dan Wu[2], See Wee Koh[1], Pingqi Gao[4], Zhichuan J. Xu[3], Sili Deng[5], Yan Zhou[2,6★], Bo You[1,7★], Hong Li[1,8,9★]

[1]School of Mechanical and Aerospace Engineering, Nanyang Technological University, 639798 Singapore

[2]Advanced Environmental Biotechnology Centre, Nanyang Environment and Water Research Institute, Nanyang Technological University, 637141, Singapore

[3]School of Materials Science and Engineering, Nanyang Technological University, 639798, Singapore

[4]School of Materials, Sun Yat-sen University, Guangzhou 510275, China

[5]Department of Mechanical Engineering, Massachusetts Institute of Technology, Cambridge, Massachusetts 02139, USA

[6]School of Civil and Environmental Engineering, Nanyang Technological University, 639798, Singapore

[7]School of Chemistry and Chemical Engineering, Huazhong University of Science and Technology, Wuhan, Hubei 430074, China

[8]Centre for Micro-/Nano-electronics (NOVITAS), School of Electrical and Electronic Engineering, Nanyang Technological University, 639798, Singapore

[9]CINTRA CNRS/NTU/THALES, UMI 3288, Research Techno Plaza, Singapore

[†]equal contribution.

[★]Correspondence to: zhouyan@ntu.edu.sg; youbo@hust.edu.cn; ehongli@ntu.edu.sg

Supplementary Notes

Cycling of the entire system. Material balance and net zero emission is the premise of a sustainable technology, so we further analyzed the recyclability of our system. We have calculated the carbon balance before and after our hybrid electrolysis and found that nearly 100% dissolved chitin was successfully converted into organic acids (Supplementary Fig. 22), which can be further verified by the GC-MS testing result (Figure 4a, b) of post-reaction sample (no other residue peaks) as well as the clear and transparent post-reaction solution. And no other chemicals, apart from milled shrimp shell and KOH electrolyte, were involved during the process. The recyclability of ball milling process can be further proved by the reusage of kaolinite (ball mill catalyst), as manifested by Yan group's previous report[1]. Hence, all the residue issues can be effectively addressed in our proposed hybrid electrolysis system. To further separate produced potassium acetate and reuse "waste KOH", we tested the pH and solution resistance (Rs) along with different mass of reactance and showed in Supplementary Fig. 17. Within the acceptable range of Rs (based on the fact that the corresponding Rs in 0.1 M KOH is around 3-4 Ω and the sharp change of Rs and pH locates at around 33 g L^{-1}), the feeding of reactance can be up to 33 g L^{-1} in 1 M KOH electrolyte. Then we used similar precipitation method as that was employed by Kanan's group[2]. The picture of collected solid was shown in the inset of Figure 3d. The quantification results of NMR and GC-FID (Supplementary Figs. 19 and 20) both show up to 99% purity of acetate hydrate. The separated potassium acetate hydrate can be further utilized in the following biological conversion to single cell protein[3, 4], while the remained potassium hydroxide solution can be used to prepare new alkaline electrolyte. The whole cycling diagram is shown in Supplementary Fig. 33.

Analysis of the electrocatalytic conversion of chitin polymer. To further prove the effective conversion of chitin long chain polymer instead of monomer, we verified the high molecular weight nature of the freeze-thawing dissolved chitin. As shown in Supplementary Fig. 23, the group of peaks locating from 2.8 to 4.0 ppm represents the non-anomeric protons on the ring skeleton[5] (the minor shift is due to the different coordination environment resulting from different pH condition), and the resolution of them are similar to that of dissolved chitosan, which further corroborates the large molecular weight nature of the dissolved chitin[6]. Besides, the MALDI-TOF-MS testing result (inset of Fig. 4d) also corroborates the polymer nature of freeze-thawing dissolved chitin. Despite the comparative low ionization efficiency and narrow detection range (only up to 40 KDa), one can clearly see the high-molecular weight nature of dissolved chitin. The dissolved chitin can be effectively converted to valuable acetate in our hybrid electrolysis (Fig. 3). However, the high molecular weight of chitin polymer also slows down the reaction rate and increases potential needed due to comparatively slow mass transportation and slow electro-depolymerization of large chitin polymer.

Possible reaction pathways from chitin monomer (NAG) to acetate and nitrate. According to the product identification and quantification measurements, we hypothesize the following reaction pathways. After the rate-determine glyosidic-bond breaking process, NAG can be dynamically hydrolyzed to open the pyranose ring and deacetylated to release HAc from the side chain. Afterward, anodically radical cleavage of C-C bond would be possible[7, 8]. Preferably, the consecutive breaking of C-C bonds at the C-3 and C5 position dominates, with the progressive release of aminoacetaldehyde, glycolaldehyde and ethenediol, because of the thermodynamic favorability and catalytic effect of the in-situ generated primary amines[9-14]. Then, the newly formed aminoacetaldehyde and ethenediol can be tautomerized to acetamide and glycolaldehyde

or acetic acid, respectively[15-17]. Afterward, acetamide can be further transformed into acetaldehyde, along with the production of nitrate[7]. While aldehydes are unstable under such a highly oxidative condition and would be oxidized into acetic acid instantly. Acetic acid is stable under the electrochemical conditions used in this work. As a result, high yield of acetic acid can be obtained.

Potential value of acetate product. Acetic acid is an important commercial organic acid with global annual production of over tens of million tons currently[18]. Besides, it also has a unique role in biosystem. It is not only the most preferable methanogen substrate, being utilized by methanogenic archaea to produce methane via biomethane production process, but also can be assimilated by bacteria and plants as fertilizer[19]. Moreover, previous study demonstrated the value of acetic acid to biosynthesize single cell protein, which can be directly utilized as animal food and organic fertilizer[3, 4]. So, the anode product, acetic acid, has great potential for bio-application, serving as an importance carbon source of protein, a unique source of nutrients for animal feeds or even human supplements. As such, this can close the loop of carbon cycle from food waste (shrimp shell from seafood waste) to food.

Scale matching between global hydrogen demanding and electrochemical valorization of chitin-based biomass feed. We propose the following main reaction equation ($C_8H_{15}NO_6 + 5OH^- \rightarrow 4C_2H_3O_2^- + 4H_2 + NO_3^-$) based on the highly selective conversion to acetic acid and nitrate from dissolved chitin. As can be seen from the equation, every 4 mole of hydrogen production needs one mole chitin monomer (NAG) feed. To meet the annual hydrogen production of 70 million tons[20], we will need chitin of 1.9 billion tons, which is less than 2% of the annual production of chitin globally [21, 22]. Thus, theoretically, chitin is a great choice that is as abundant as water to support hydrogen economy. The comparatively low solubility of raw biomass, arising from their rigid polymeric structures, has greatly hindered the development of raw biomass

reforming. While recent progress in mechanochemical amorphization greatly improved the processibility of raw biomass. Specifically, our mechanochemical pretreatment can realize more than 90% solubility of chitin.

Moreover, understanding the extraction technology of chitin is not mature yet, and that results in the high cost to fractionate chitin, we thus extended our technology to raw chitin-containing biomass, shrimp shell that can be readily extracted from seafood waste (current market price < USD 0.1 kg^{-1}). Delightfully, shrimp shell waste exhibits similar processability via our hybrid electrolysis after mechanochemical pretreatment. Other raw chitin-containing biomass (e.g., fungus) can be even more readily processed since shrimp shell is one of the most mechanically strong chitin-containing biomass. Notably, the advances of technology to extract chitin from natural biomass can further increase the viability of our hybrid electrolysis.

Besides, the anodic product, acetic acid, not only is an important platform chemical, but also has unique role in biosystem. It has great value to the synthesis of single-cell protein, which may serve as animal feed, and thus closes the loop of food waste. Therefore, the scale of electrochemical valorization of chitin-based biomass feed could readily meet the global hydrogen demanding.

Thereafter, the scale of electrochemical valorization of chitin-based biomass feed can fully meet the global hydrogen production demanding.

Brief energy efficiency analysis. The total energy balance is challenging to be calculated precisely at this stage because the energy cost in maintenance such as pump, drier, gas purifier (an energy intensive process) varies from equipment to equipment.[23] Instead, one could make a direct comparison with that the state-of-the-art AWE, as shown in Supplementary Table 4. There are many factors affecting the energy efficiency. For instance, water purification and heating in AWE, and system restart at 2% H_2 crossover during operation.[24] On average, AWE has an energy efficiency up to 80%.[25] In comparison with AWE, our hybrid electrolysis consumes about 0.17 V less voltage at the same current density of 0.2 A cm^{-2}, suggesting up to 10% higher efficiency, without considering the energy consumption in biomass pretreatment.

Next, we discuss the energy consumption in pretreatment of biomass. The energy consumption in ball mill treatment depends on the source and type of biomass[26]. A further reduction of energy consumption is possible by using the right catalysts and/ or increasing the processing scale. Inspired by previous work on oxidant-catalyzed decomposition of lignocellulose [27-29], we added oxidizing agent H_2O_2 in ball milling pretreatment step to accelerate decomposition of chitin polymer. Interestingly, with only 3 wt% H_2O_2 solution added, the ball mill time can be decreased from 6 h to 2 h, with almost the same dissolution effect, as shown in new Fig. 31 and 32. Accordingly, the energy efficiency can be greatly improved with the help of catalysts during the ball milling pretreatment. As a result, the overall energy consumption can be greatly reduced.

Nevertheless, with current ball mill energy of 950 MJ kg^{-1} H_2, the total cost of H_2 produce is comparable to that of AWE already because of the much more valuable anodic product (acetate) compared to that of AWE (oxygen), as shown in Supplementary Table 5. Therefore, with improved efficiency of ball mill pretreatment, we will make H_2 production cheaper than that of the state-of-the-art AWE. A direct energy efficiency comparison between our hybrid electrolysis and the state-of-the-art AWE is shown in Supplementary Table 6 and 7.

Supplementary Figures

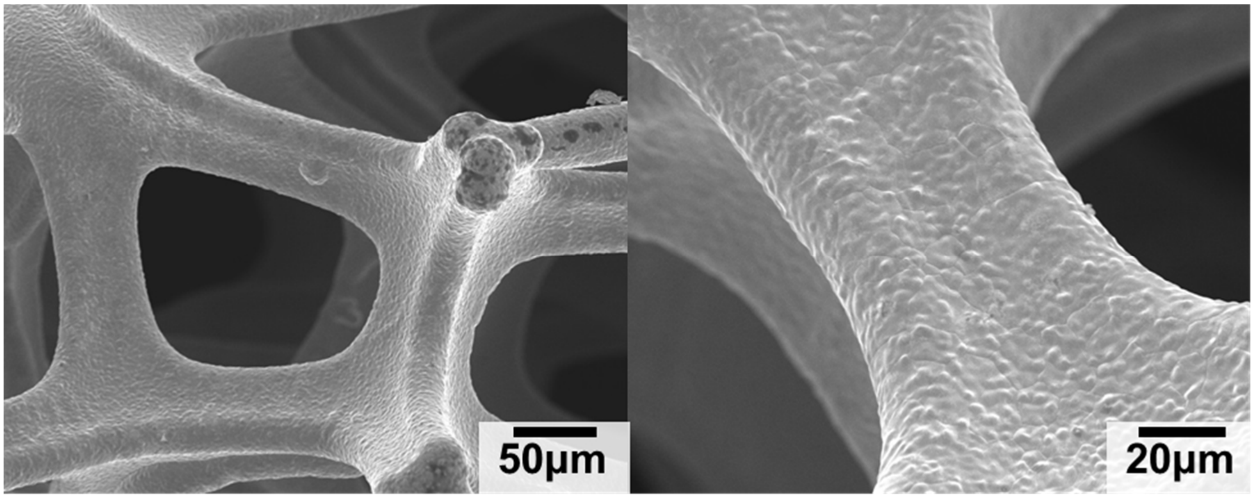

Supplementary Figure 1| Morphology of Ni form. SEM images of Ni foam substrate at different magnifications. 3D porous structure with smooth surface

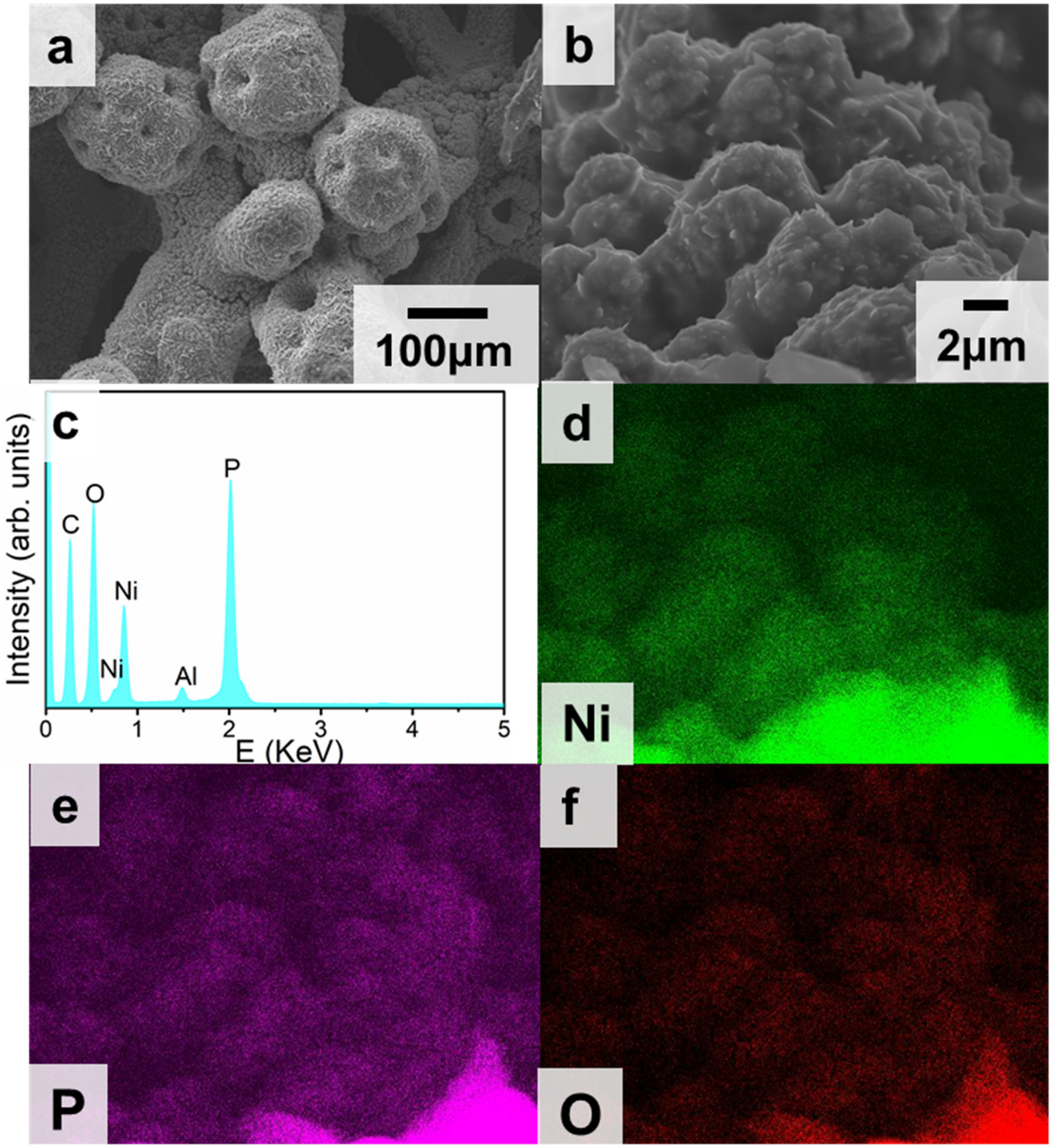

Supplementary Figure 2| Material characterization of Ni_2P/ NF. a, SEM images of a Ni_2P/ NF sample. **b**, Zoom-in view of **a**, shows the urchin like surface. **c**, EDS spectrum of the area shown in (**b**). **d-f**: Ni, P and O element mapping.

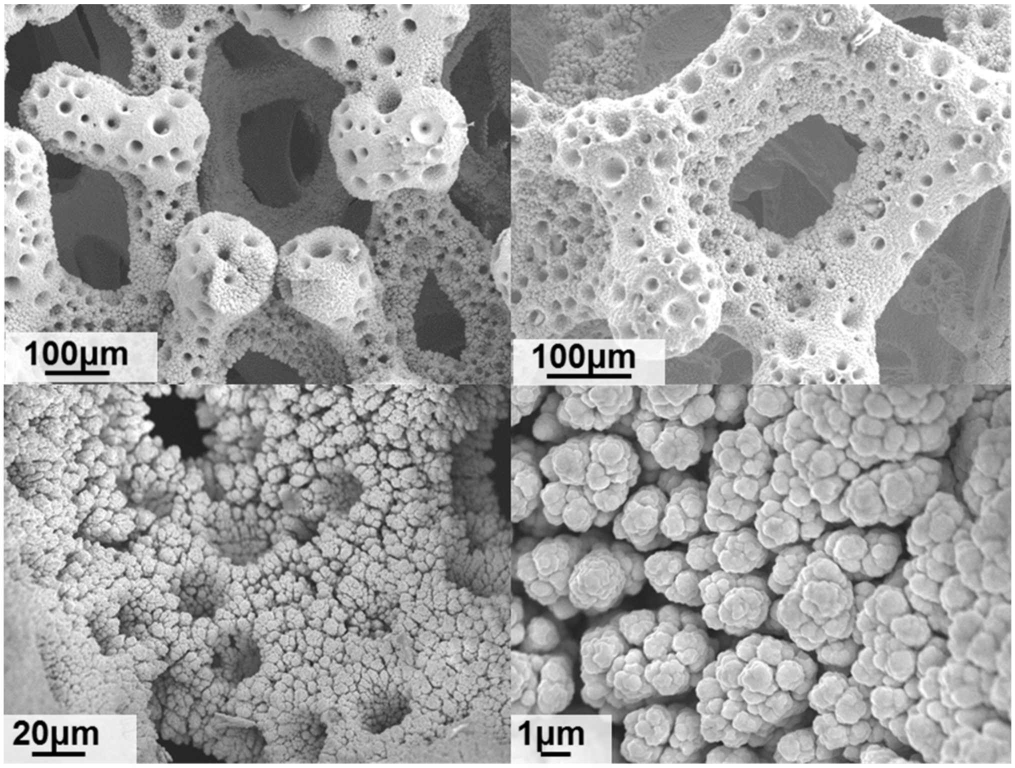

Supplementary Figure 3| SEM images of *hp*-Ni anode. It shows the pore size range from several micrometer to hundreds of micrometers.

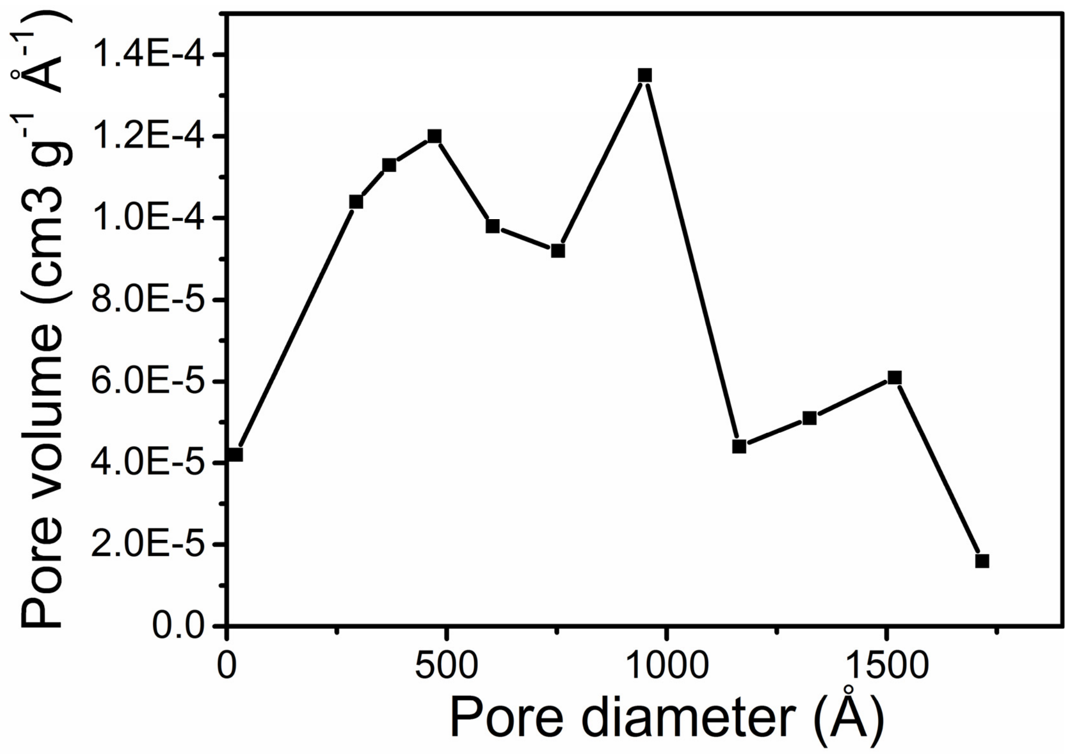

Supplementary Figure 4| BJH adsorption pore distribution. Well-defined large pore size distribution was observed. To quantify the pores with even lower dimensions than those observable in SEM, Brunauer–Emmett–Teller (BET) isothermal nitrogen adsorption measurement was done to show pore size distribution below 200 nm.

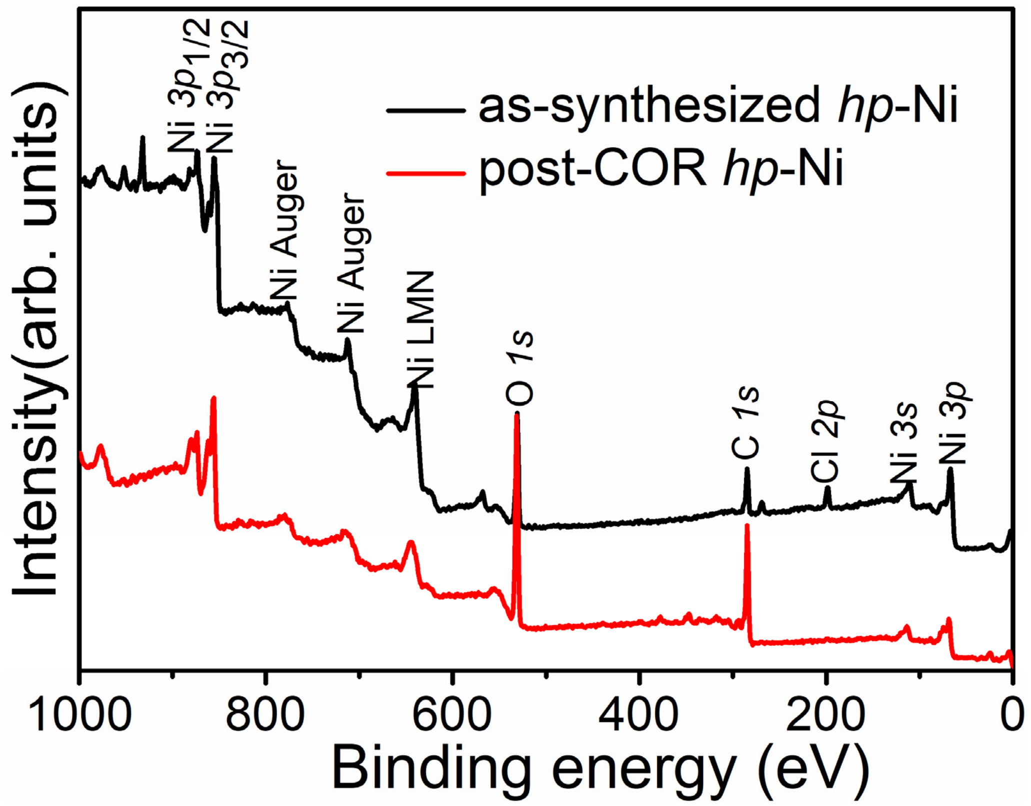

Supplementary Figure 5| XPS survey spectrum of as-synthesized and post-COR *hp*-Ni samples. XPS survey shows that there is no other element shown in fresh and post-COR *hp*-Ni samples other than Ni and O.

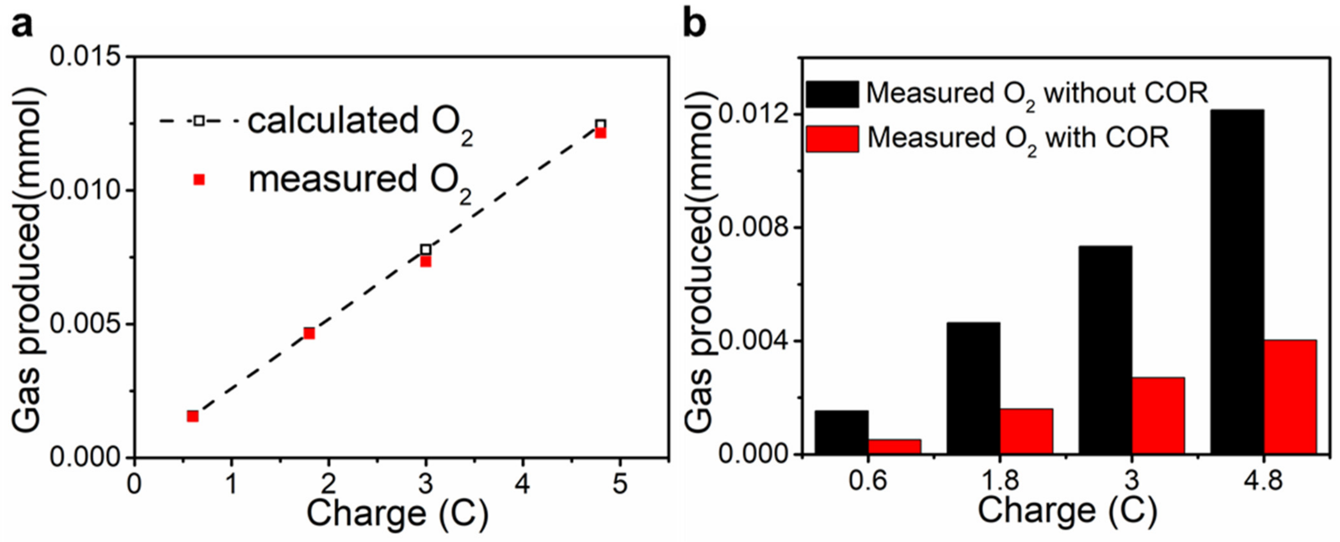

Supplementary Figure 6| Identification and quantification of gas product. a. GC-FID quantification of oxygen product without adding chitin at constant potential of 1.6 V vs. RHE. **b**, Comparison of measured oxygen with and without adding of 33.3 mg L^{-1} chitin at constant potential of 1.6 V vs. RHE.

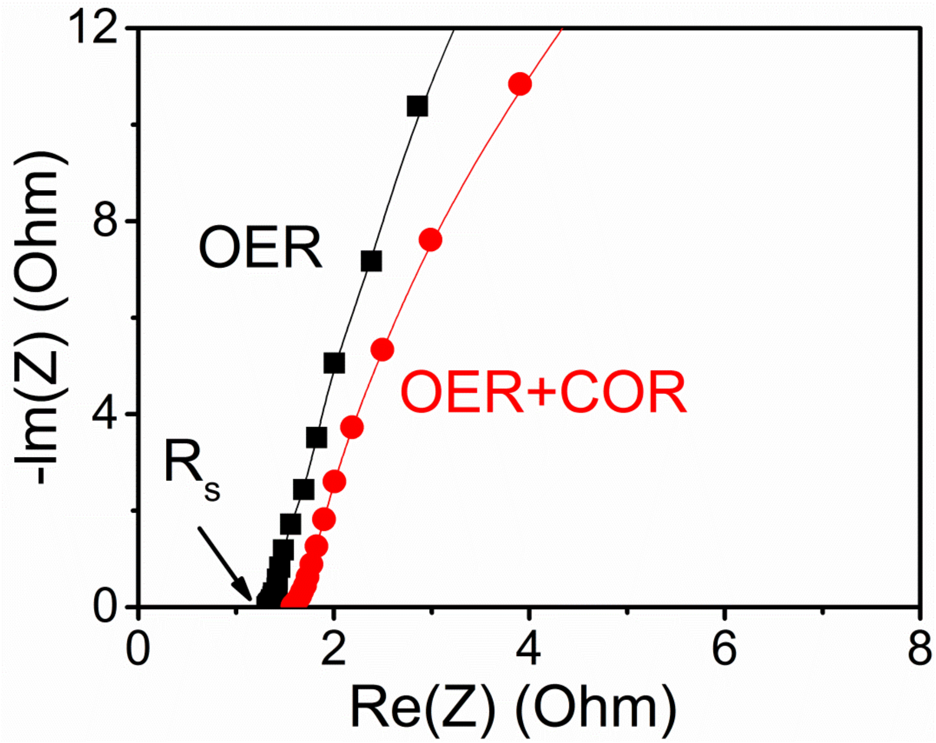

Supplementary Figure 7| Electrochemical impedance spectra. Room in view of the Nyquist plot in the inset of Fig. 3b to show the Rs.

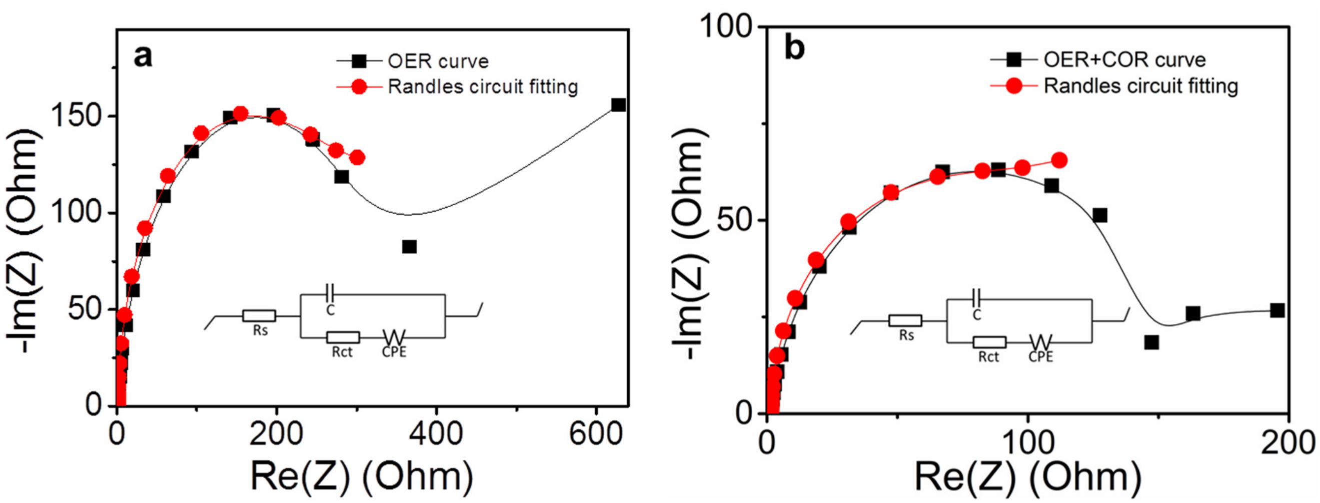

Supplementary Figure 8| Electrochemical impedance spectra at the open circuit. Nyquist plots of **a,** OER and **b,** COR+OER. The equivalent Randles circuits are inserted.

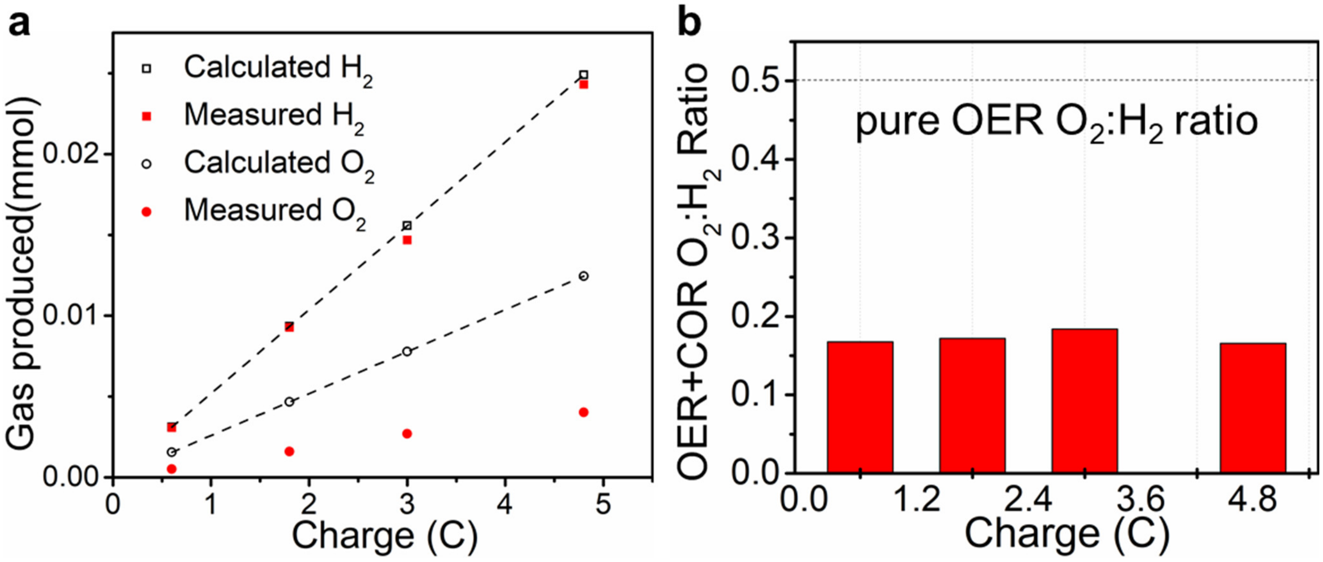

Supplementary Figure 9| Identification and quantification of gas product. a, Experimental H_2 and O_2 quantity of hybrid COR and HER electrolysis comparing with the theoretical quantity at constant potential of 1.6 V vs. RHE. **b**, The ratio of O_2/ H_2 at different reaction time for hybrid COR and HER electrolysis in (**a**).

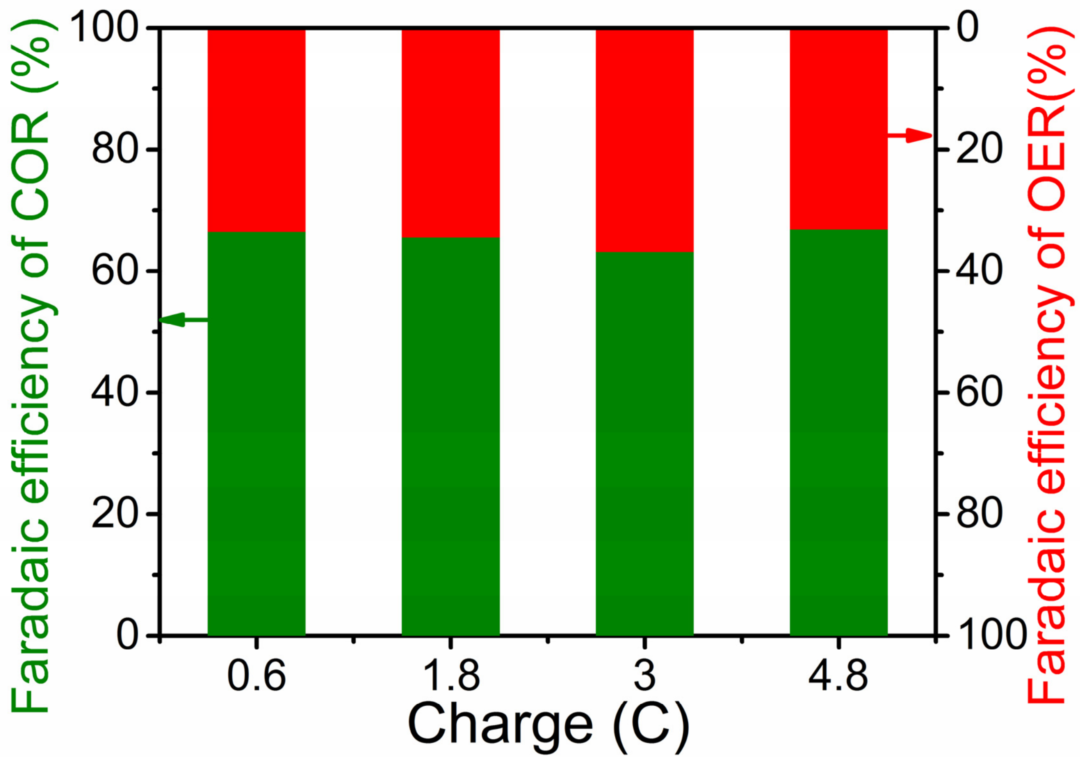

Supplementary Figure 10| Faradaic efficiency. The faradaic efficiency of COR and OER for the hybrid electrolysis under 33.3 mg L^{-1} chitin added at constant potential of 1.6 V vs. RHE.

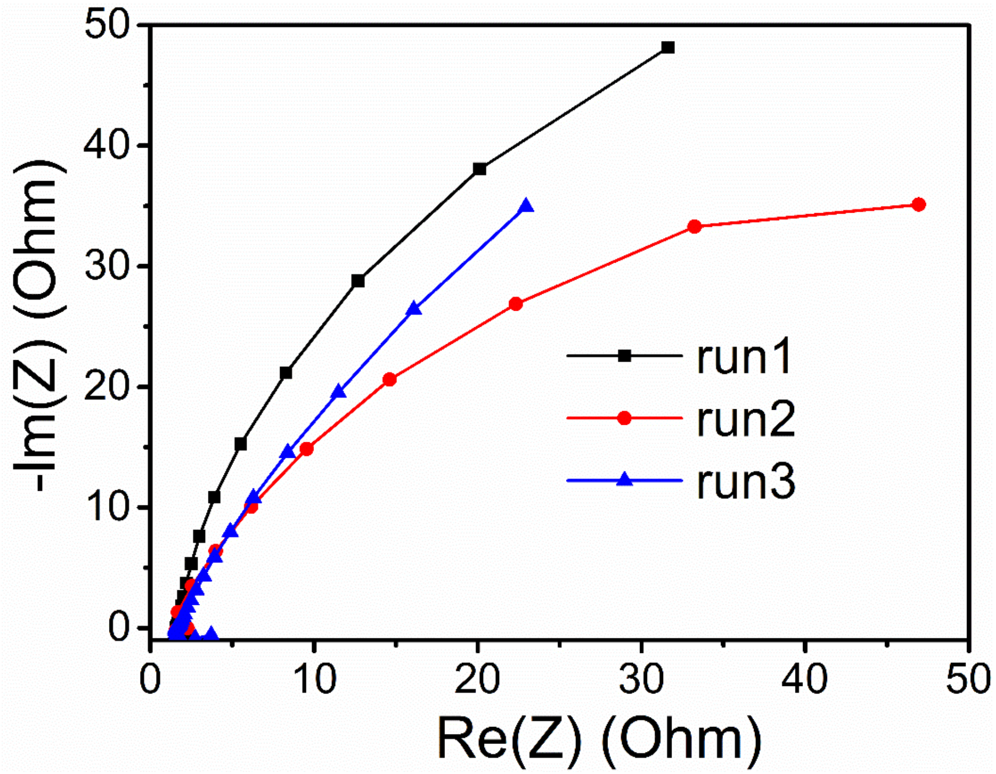

Supplementary Figure 11| EIS spectra. EIS at open circuit potential for consecutive three runs of chitin oxidation reaction (COR) in 1.0 M KOH with 33.3 mg L^{-1} chitin in Fig. 3c and the inset.

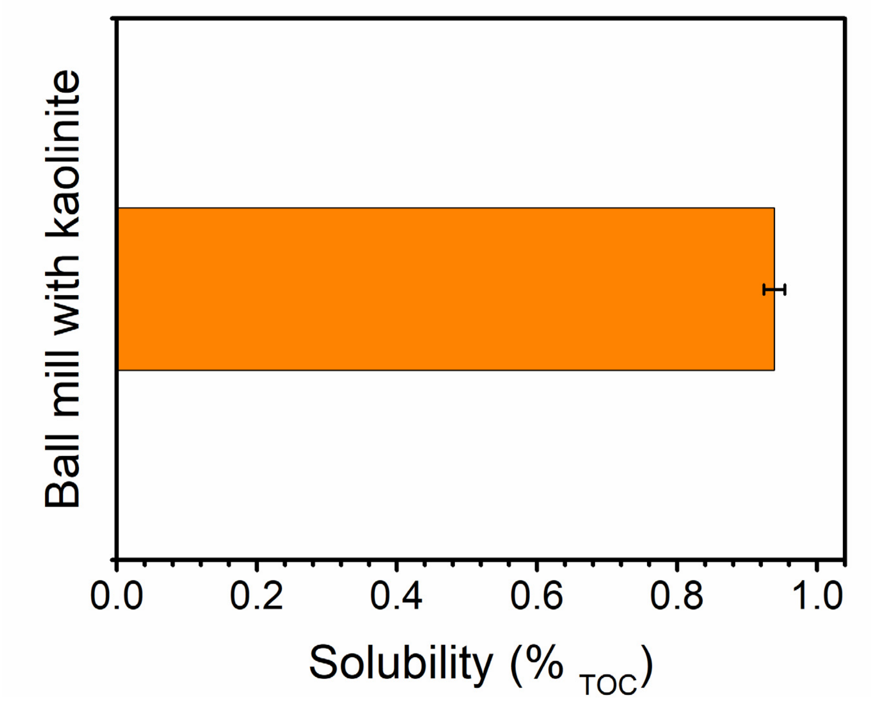

Supplementary Figure 12| Solubility enhancement by mechanochemical pretreatment. TOC based solubility test. Error bar stands for standard deviation and more than four samples were collected.

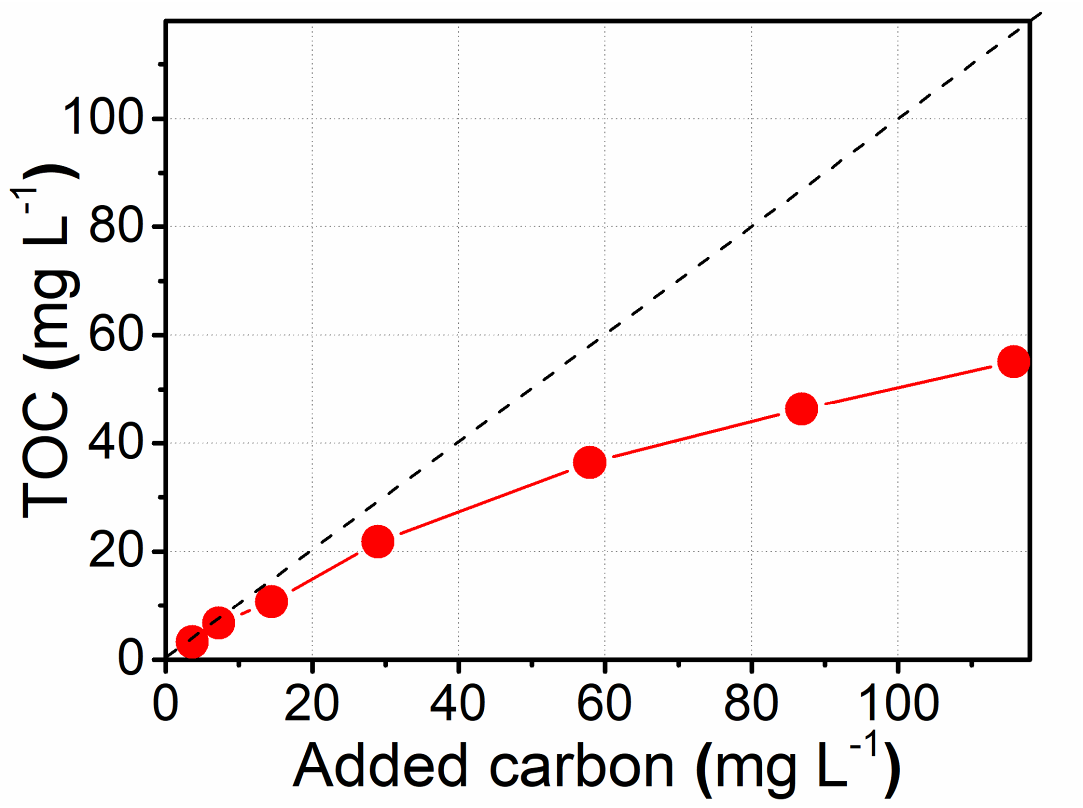

Supplementary Figure 13| Solubility of chitin via freeze-thawing method. Dissolution curve of freeze-thawing-method dissolved chitin in 1.0 M KOH solution by TOC testing. 45-degree dotted line shows 100% solubility.

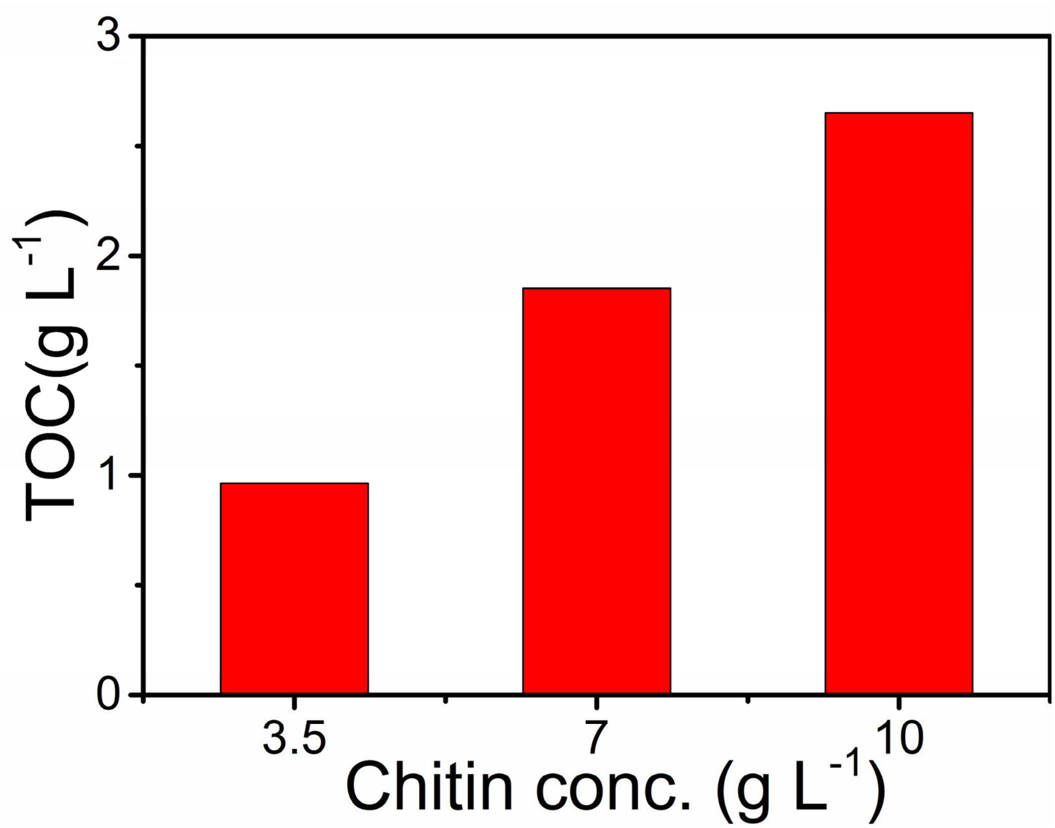

Supplementary Figure 14| Solubility of chitin via mechanochemical pretreatment. TOC histogram of M-chitin samples with different mass added in each run.

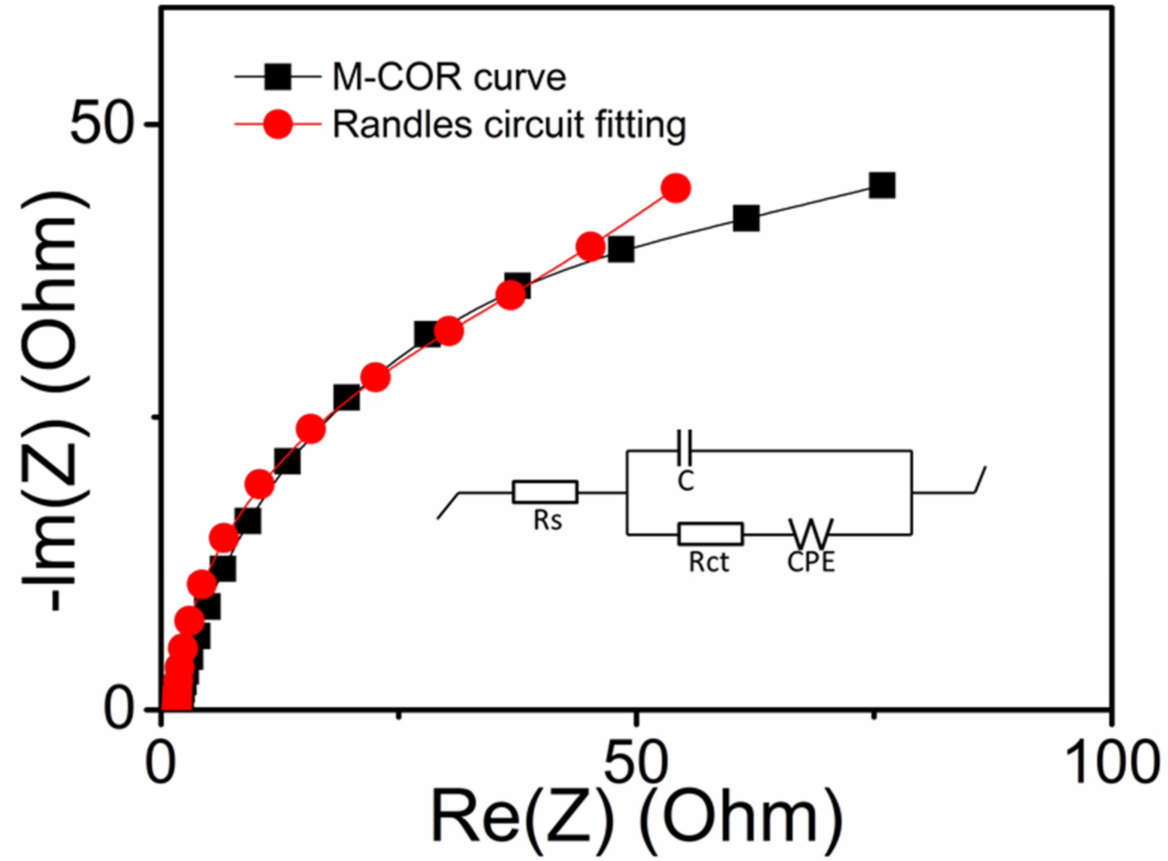

Supplementary Figure 15| Electrochemical impedance spectra at the open circuit potential.
Nyquist plots of M-COR with Randles circuit fitting.

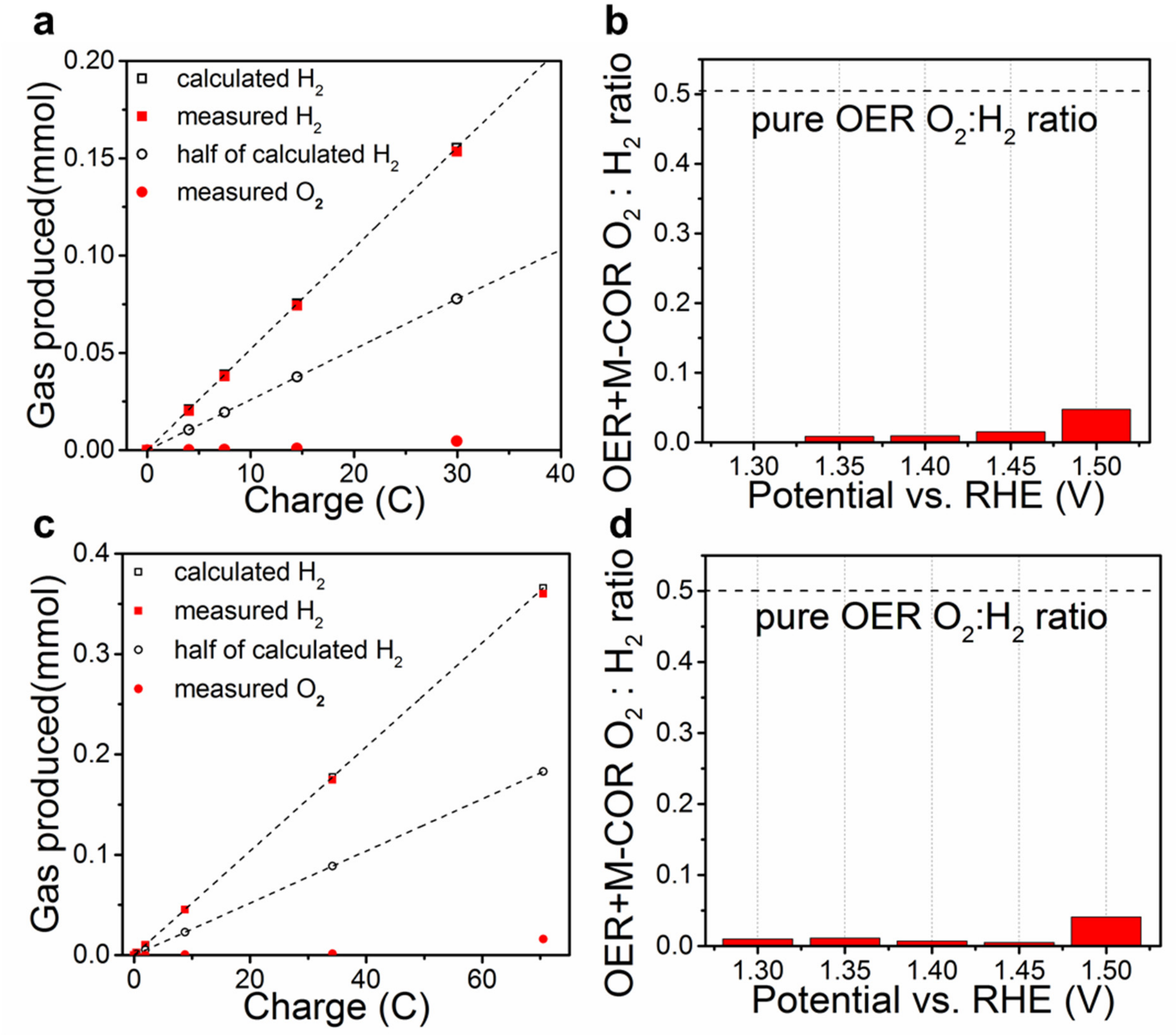

Supplementary Figure 16| Identification and quantification of gas product. a, Experimental H_2 and O_2 quantity of hybrid M-COR and HER electrolysis comparing with the theoretical quantity at different potentials. **b**, The ratio of O_2:H_2 at different at different potentials for hybrid M-COR and HER electrolysis in (**a**). **c**, Experimental H_2 and O_2 quantity of hybrid NOR and HER electrolysis comparing with the theoretical quantity at different potentials. **d**, The ratio of O_2: H_2 at different potentials for hybrid NOR and HER electrolysis in (**c**). Charge accumulation is calculated in chronoamperometric measurement for 2 mins.

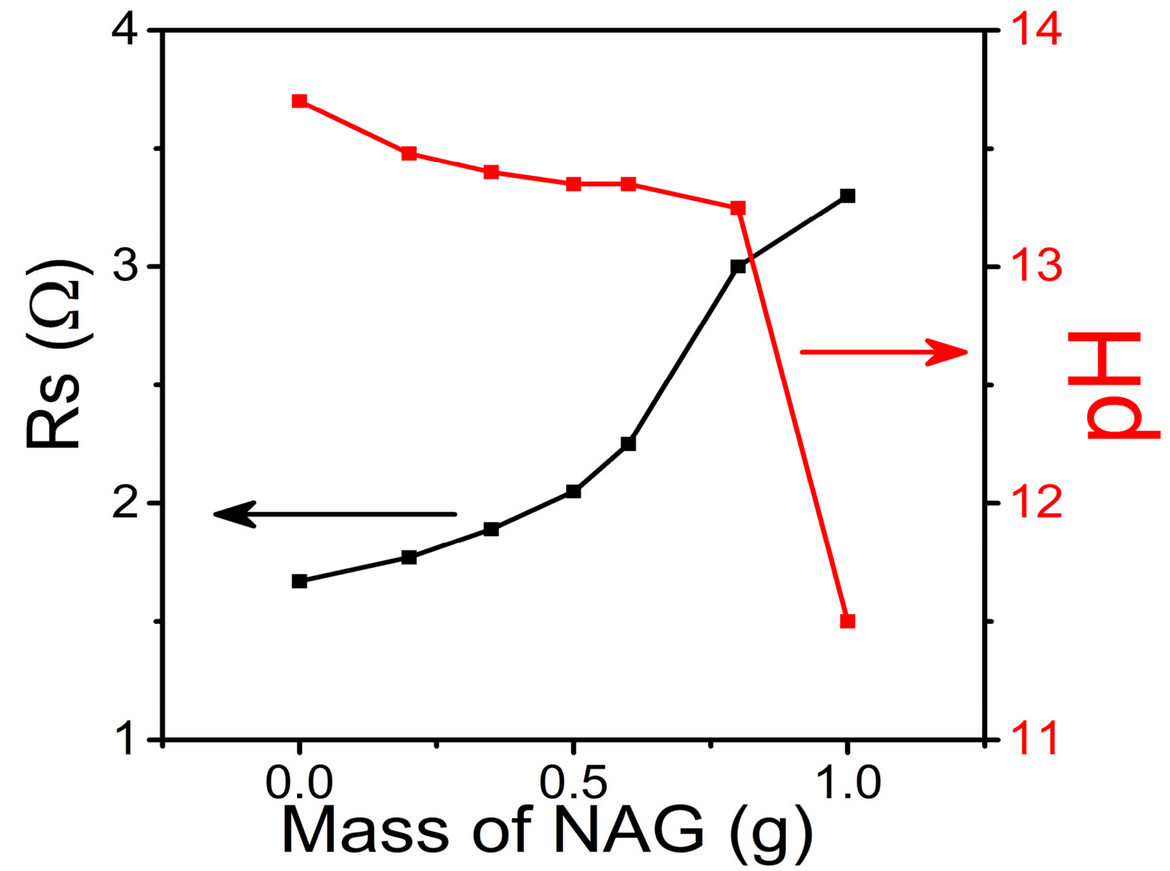

Supplementary Figure 17| Upper limit of the concentration of NAG. Dependence of electrolyte resistance (Rs) and pH value on the amount of reactant added. The electrolyte was 30 mL 1.0 M KOH solution.

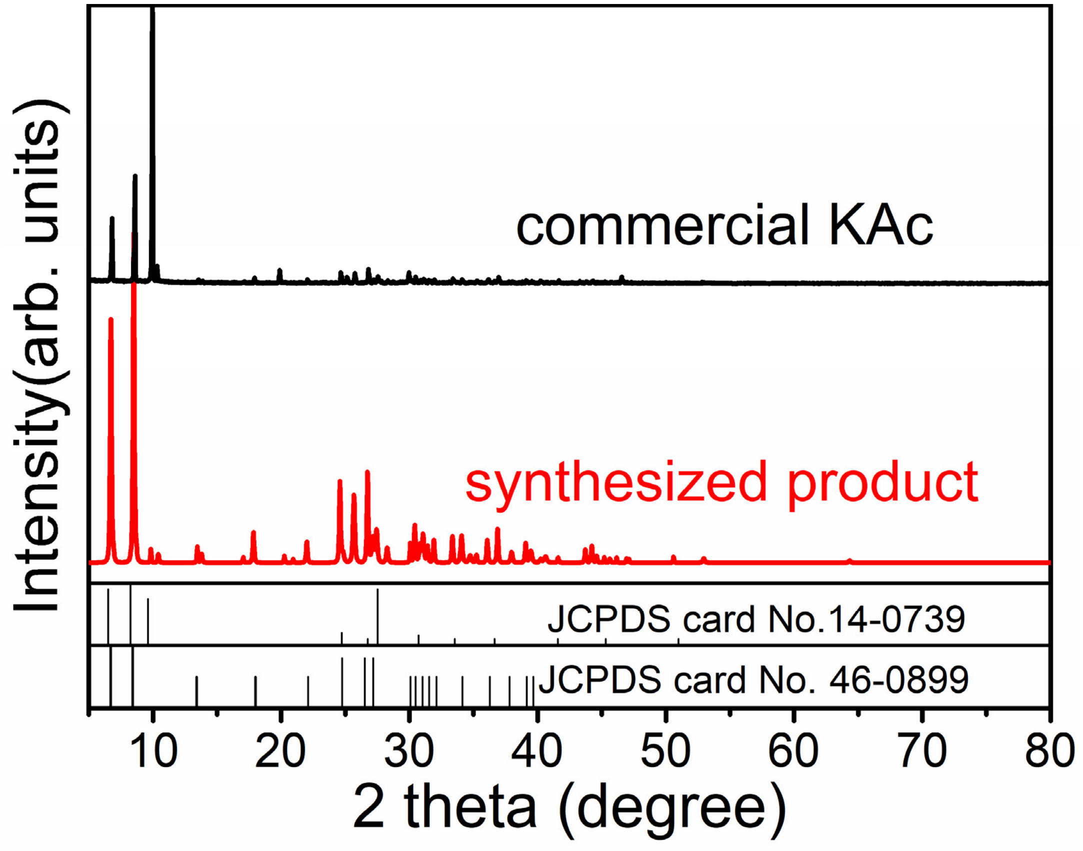

Supplementary Figure 18| Identification of collected crystal solid. XRD pattern of collected crystal product. Commercial potassium acetate is shown for comparison.

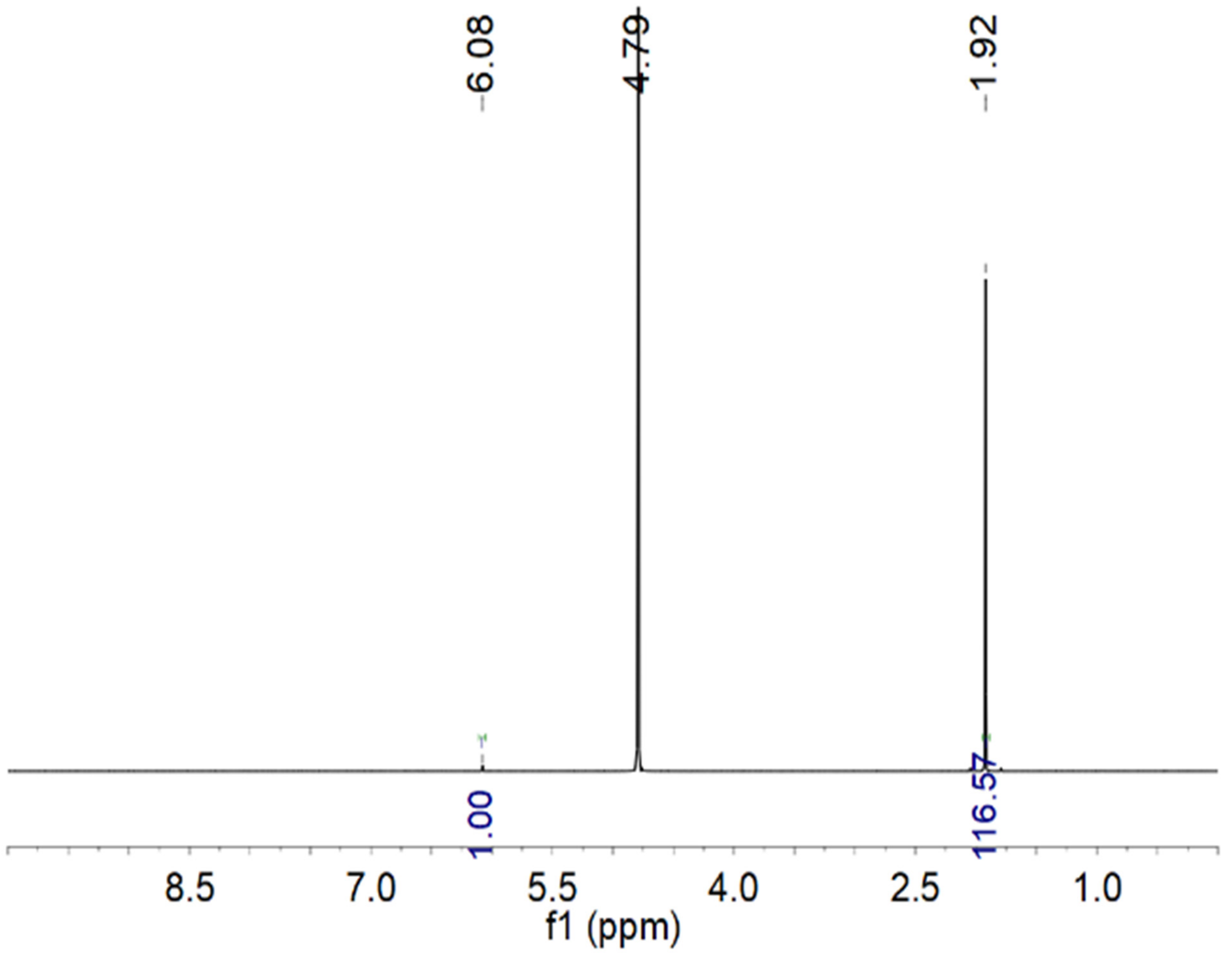

Supplementary Figure 19| ¹H NMR spectra of collected solid crystal product. 0.2 ml of 25 mM Maleic acid (peak signal at 6.08 ppm) was added as a quantification standard.

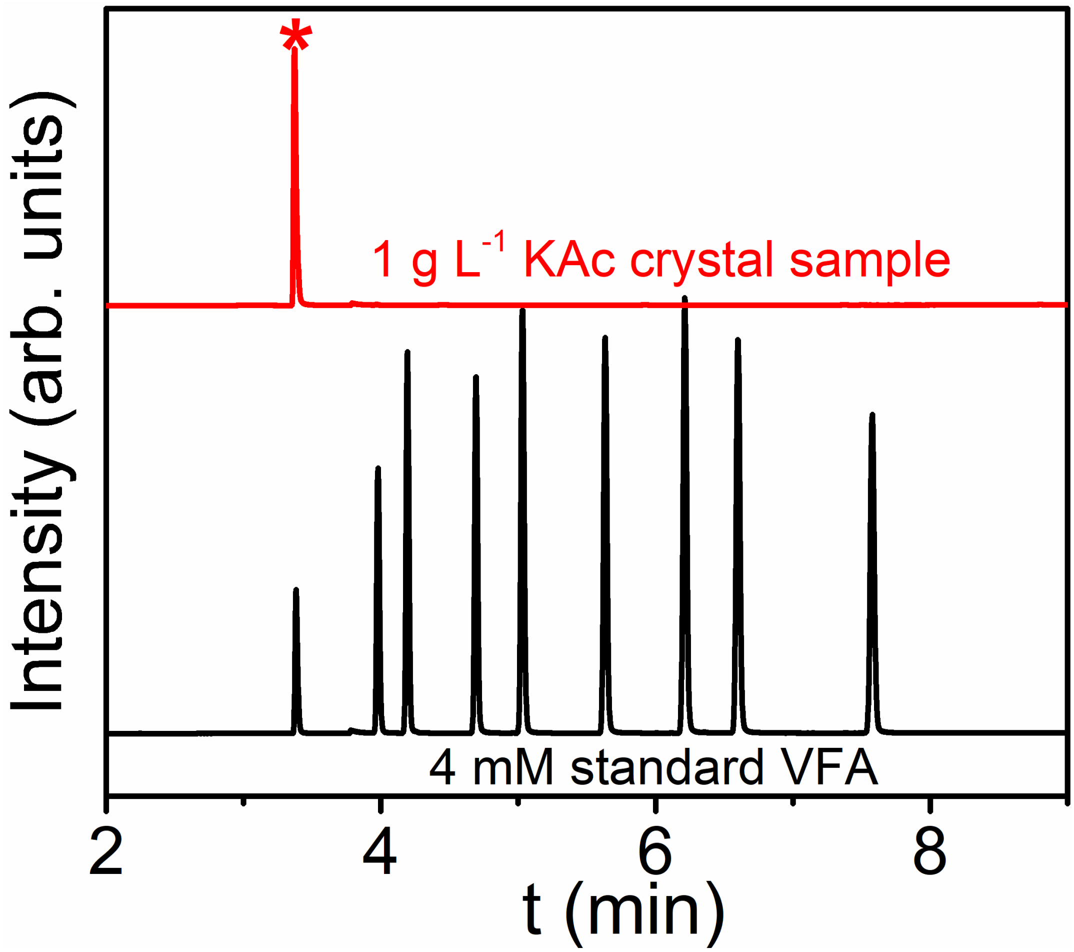

Supplementary Figure 20| GC-FID quantification of collected acetate crystal products. The spectrum of standard VFAs sample is shown for comparison.

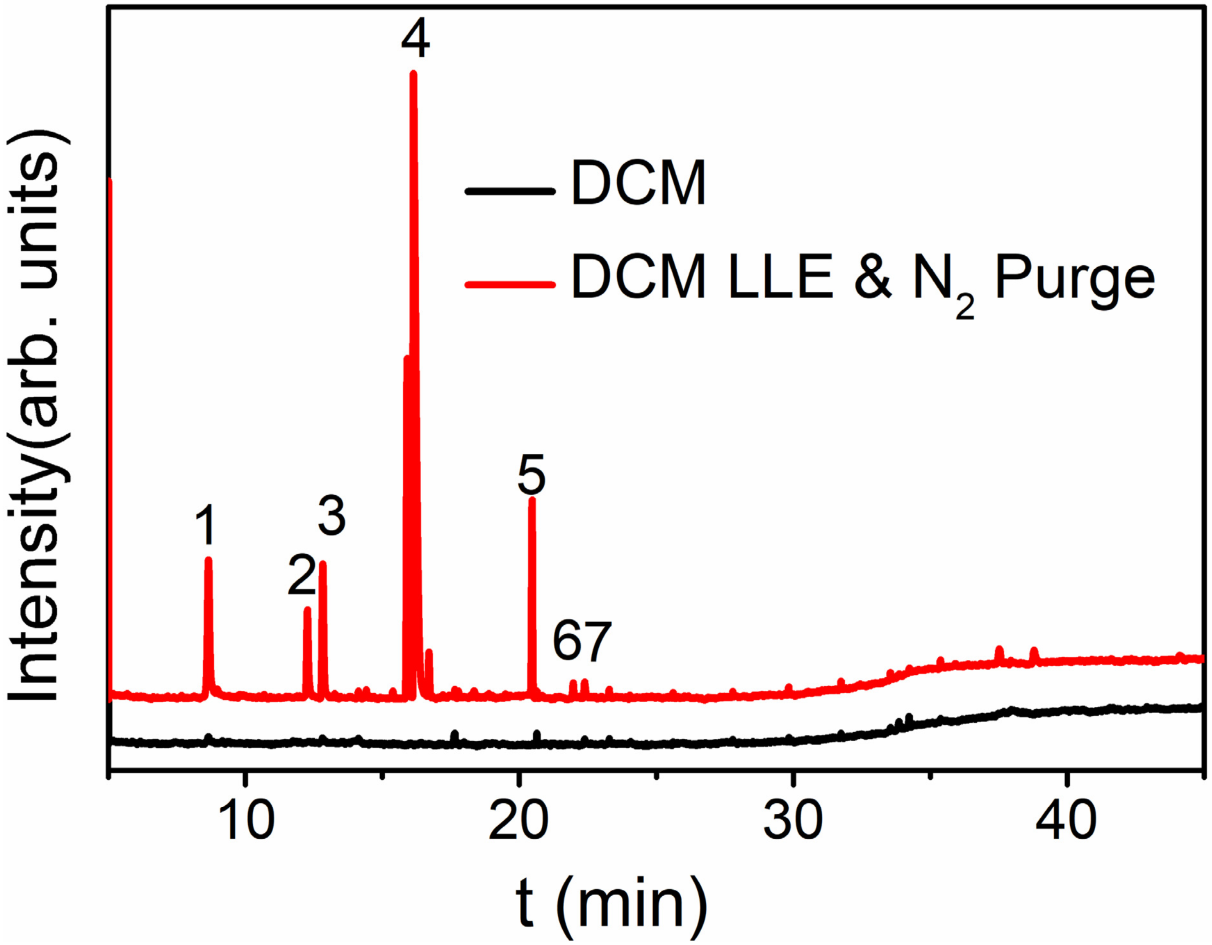

Supplementary Figure 21| Identification of the intermediates of COR. GC-MS measurement of COR intermediates using dichloromethane liquid-liquid extraction with nitrogen purge and capture. The corresponding chemicals of the peaks are (1) Tetrahydrofurfuryl alcohol, (2) 2-Hydroxyisobutyric acid, (3) 3-hydroxybutyraldehyde, (4) Acetic Acid, (5) 5-Methyl-3-hexanol, (6) 3-Amino-2-methylpropanoic acid, and (7) Acetamide. In addition, 2-butenedioic acid, glyoxalic acid and threitol were identified by methanol SPE.

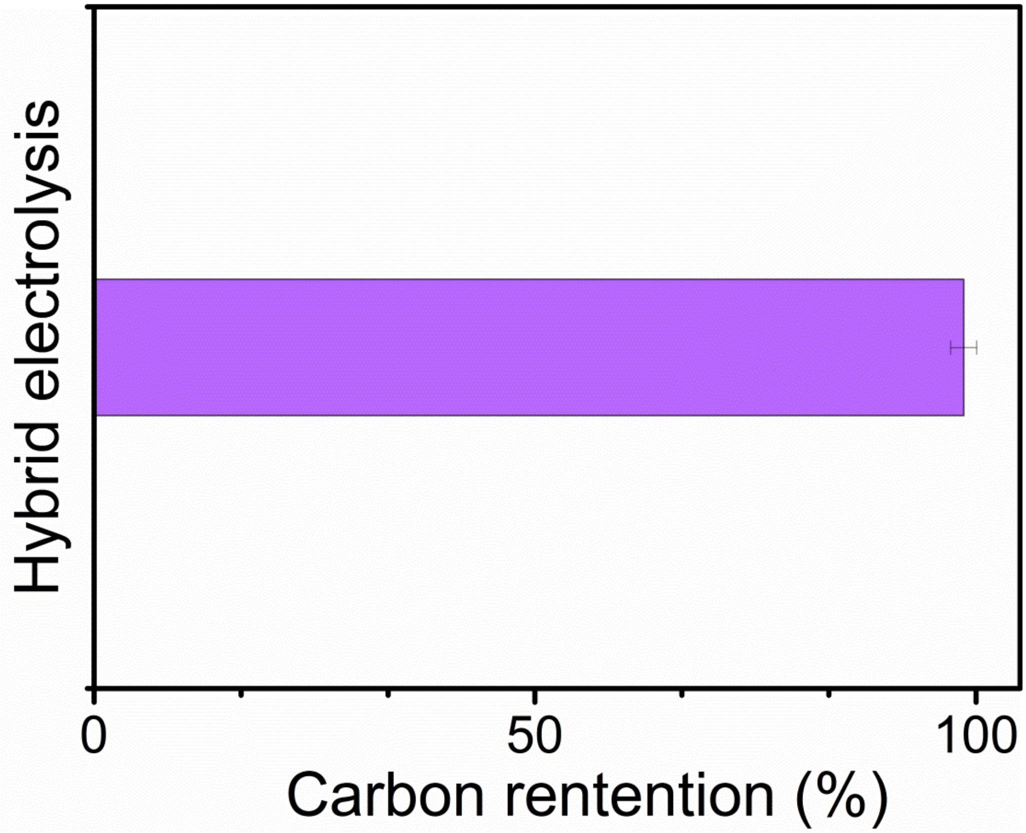

Supplementary Figure 22| Carbon balance during the hybrid electrolysis. Calculated carbon retention by the ratio of carbon in the products to that in the reactants. Error bar stands for standard deviation and more than four samples were collected.

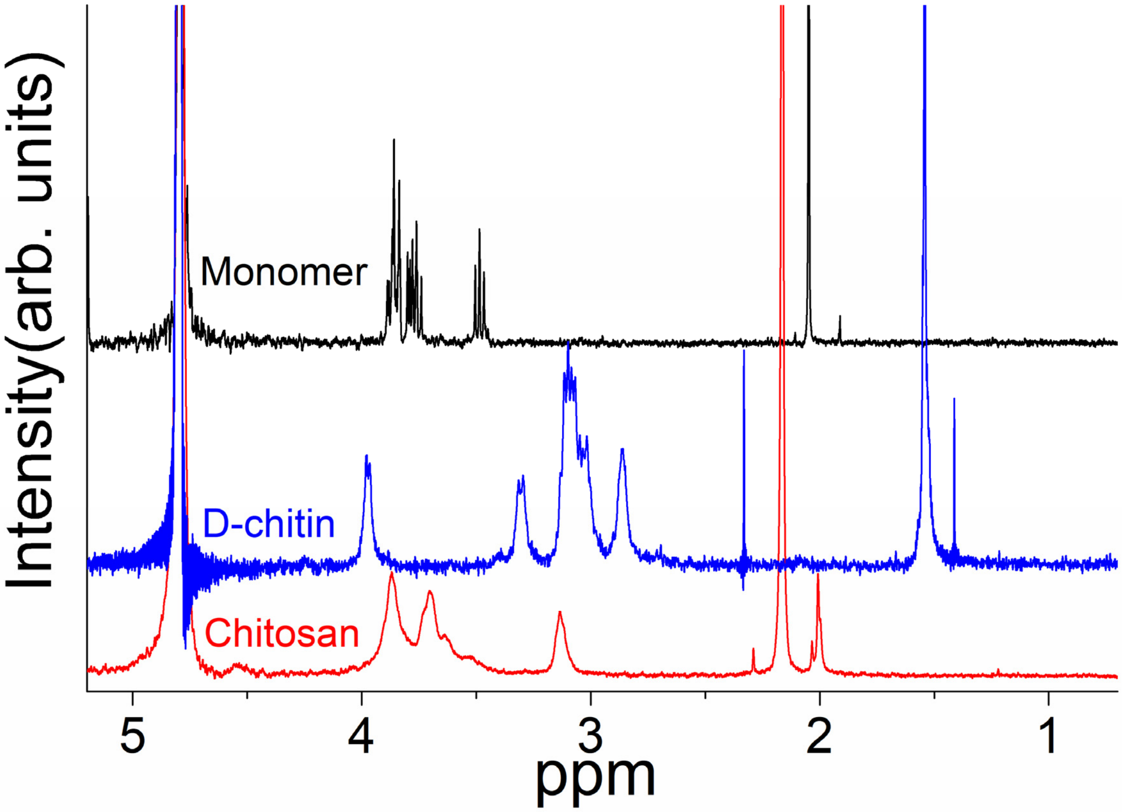

Supplementary Figure 23| Characterization of molecular weight via NMR. ^{1}H NMR spectra of acidified commercial chitosan, dissolved chitin and monomer (NAG) dissolved in D_2O solution.

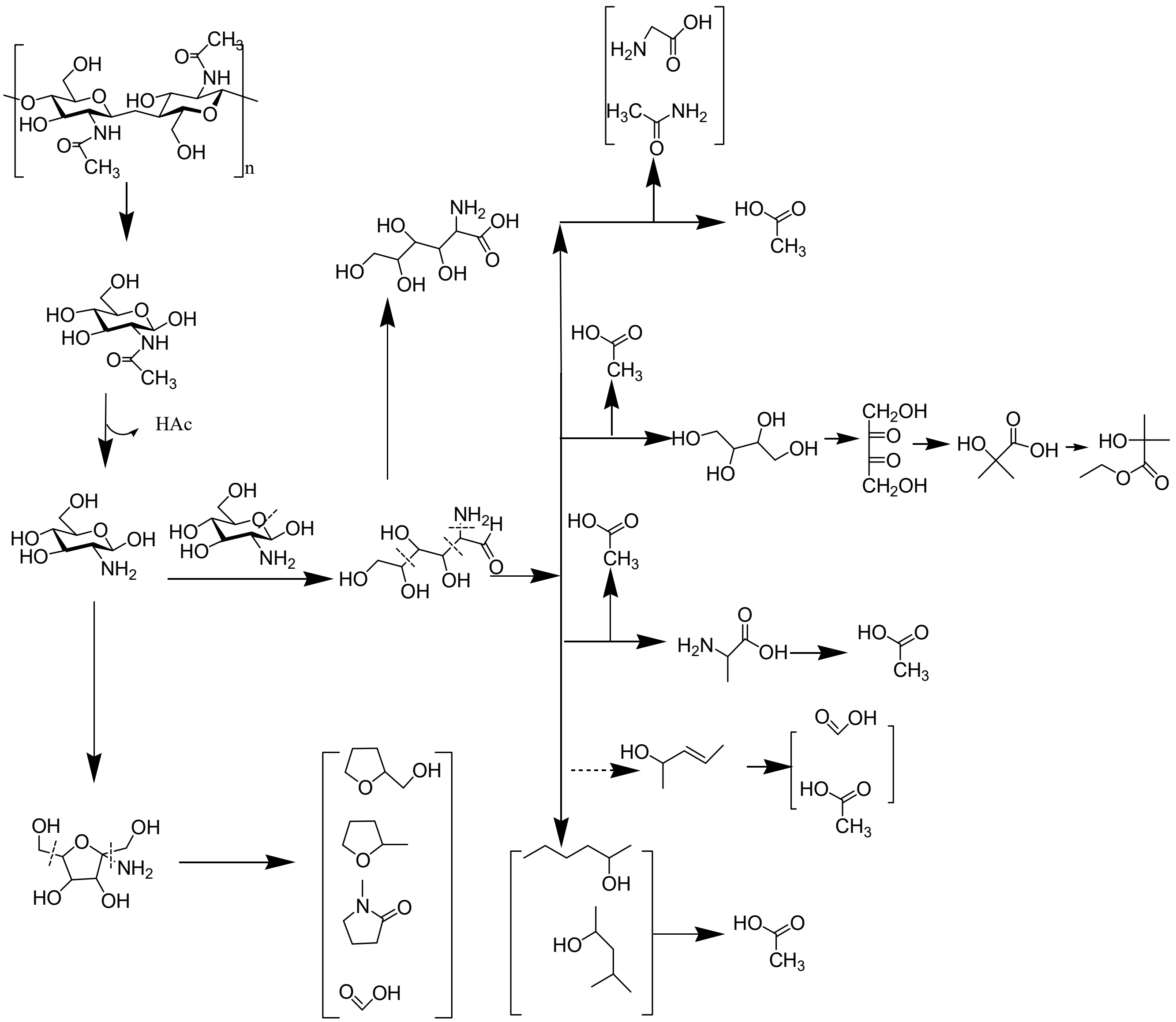

Supplementary Figure 24| Proposed pathways for COR. Possible reaction pathways from chitin polymer to main acetic acid product based on detected intermediates and products. Up to nine intermediates were identified in this work.

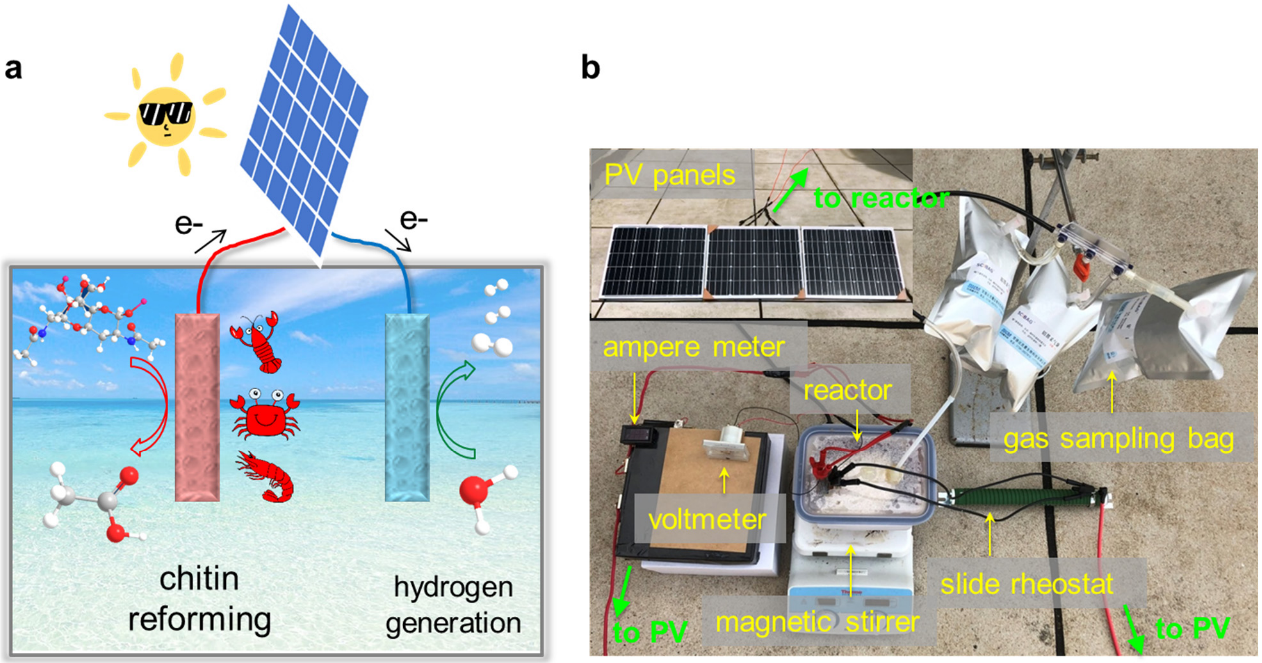

Supplementary Figure 25| Solar-drive single-compartment hybrid electrolysis. a, Schematic setup, **b**, actual setup. Commercial PV panels were used to drive the electrolyzer. A customized sealed reaction cell was connected to 3 PV panels in parallel (maximum power of 50 W each) through a slide rheostat (0-1 Ohm). A voltmeter and ampere meter are connected to the circuit to read the real-time fluctuating electrical signal in the circuit. 4 gas sampling bags were used to collect the gaseous product in 1 h reaction duration. The initial reactant (milled chitin) concentration was 5 g L^{-1}. Inset: 3 PV panels connected in parallel.

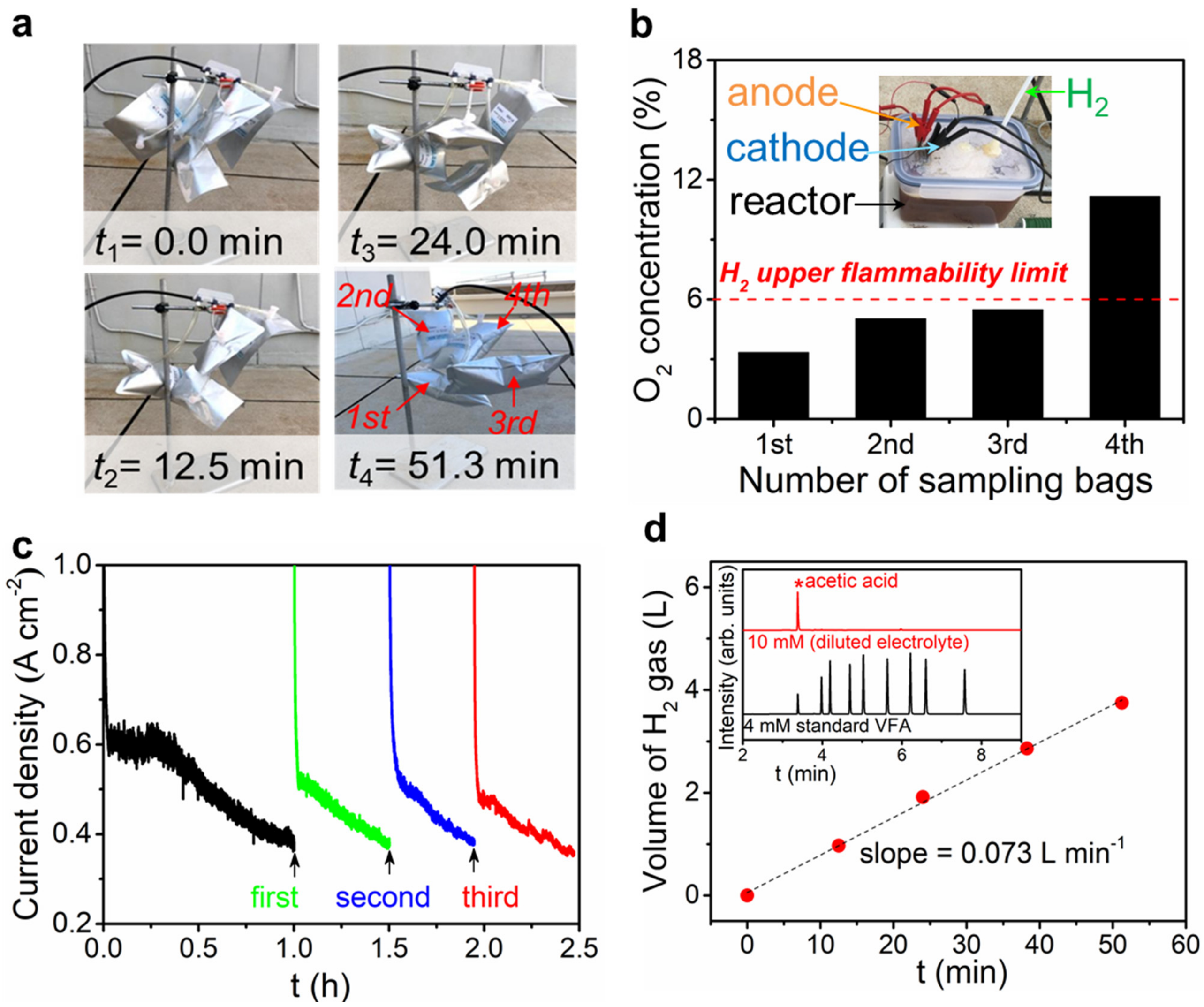

Supplementary Figure 26| Solar energy-driven single-compartment hybrid electrolysis. a, Optical images of gas collected at different time in 4 sampling bags with capacity of 1 L each. A total of 4 L gases are collected in 51.3 mins. The numbers of sampling bags are labelled (see Supplementary Fig. 25 and Movie 1). **b**, The O_2 concentration of the gaseous product in the sampling bag 1-4 collected sequentially. O_2 concentration of 6% corresponds to H_2 upper flammability limit in H_2-O_2 gas mixture (red dashed lines), above which the gaseous product is explosive. Inset: optical image of the homemade sealed reactor (see Supplementary Fig. 25 for full setup). **c**, Current density versus reaction time for 4 consecutive addition of 5-mL milled chitin to the original 25-mL milled chitin (3.5 g L^{-1}). The chitin oxidation current leaps whenever new chitin solution is added. **d**, H_2 production rate (0.073 L min^{-1}). Inset: GC-FID quantification of HAc of diluted (5 times) electrolyte.

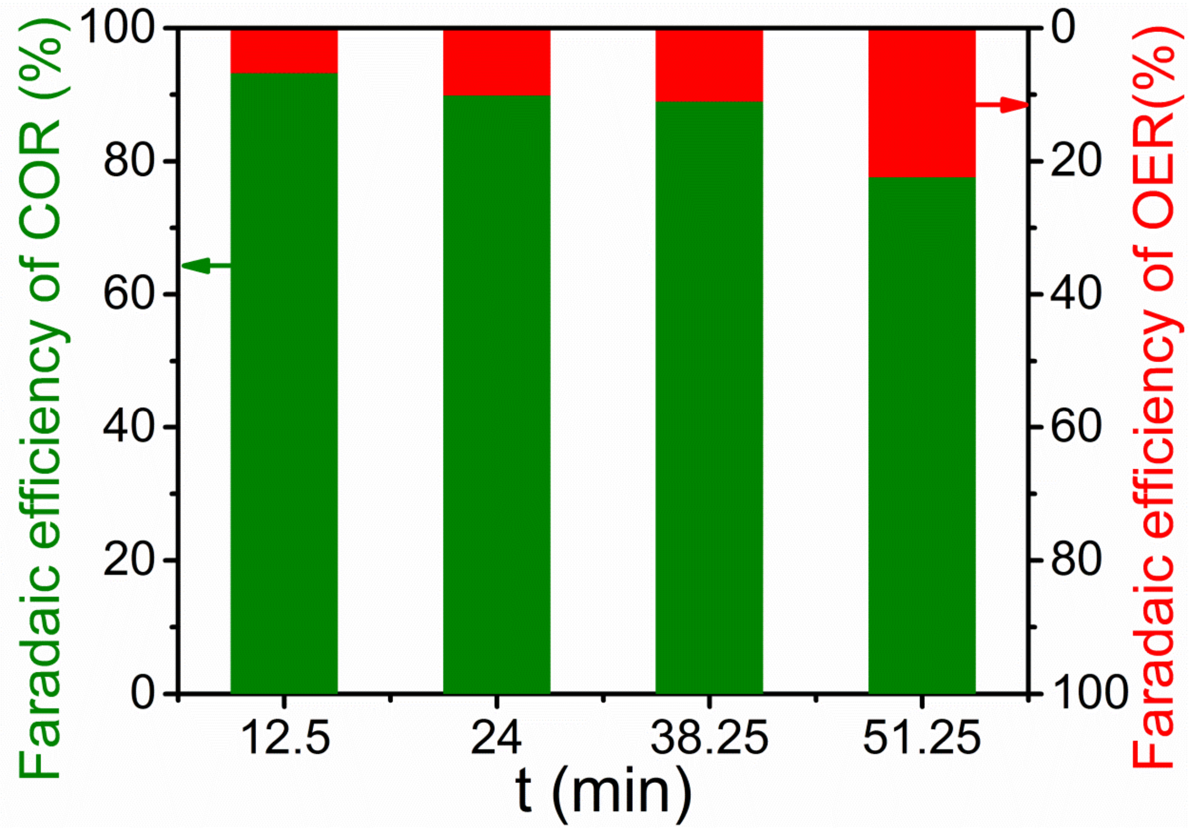

Supplementary Figure 27| Faradaic efficiency. Faradaic efficiency of COR and OER for the solar energy-driven single-compartment hybrid electrolysis at different reaction time in Supplementary Fig. 26.

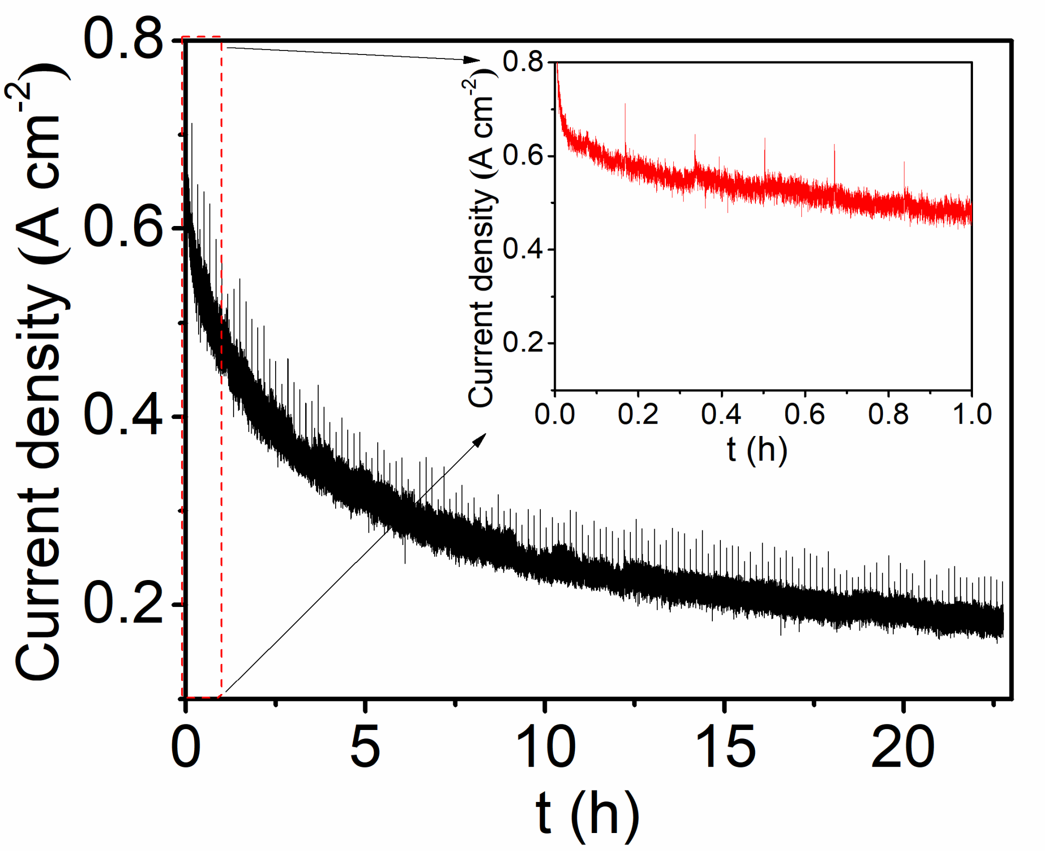

Supplementary Figure 28| Stability test of COR. The current density versus reaction time for over 20 h with initial reactant (milled chitin) concentration of 3.5 g L^{-1}. Inset: the current density evolution in the first hour of reaction.

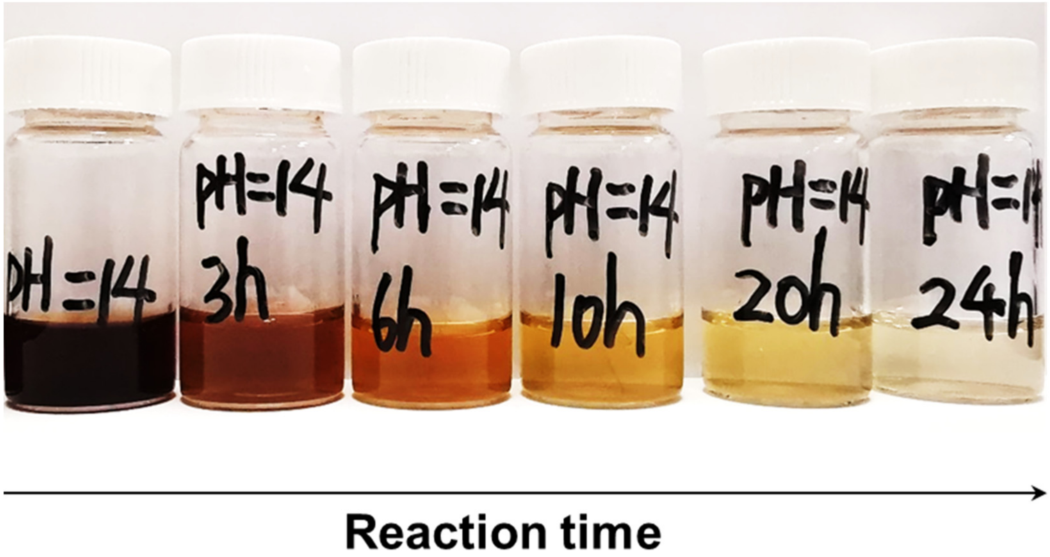

Supplementary Figure 29| Oxidation of milled chitin. The optical images of milled chitin (initial concentration of 3.5 g L^{-1}) after oxidation reaction of durations, t=0 (as prepared), 3h, 6h, 10h, 20 h, and 24h from left to right. The color of the solution became clearer as oxidation progressed.

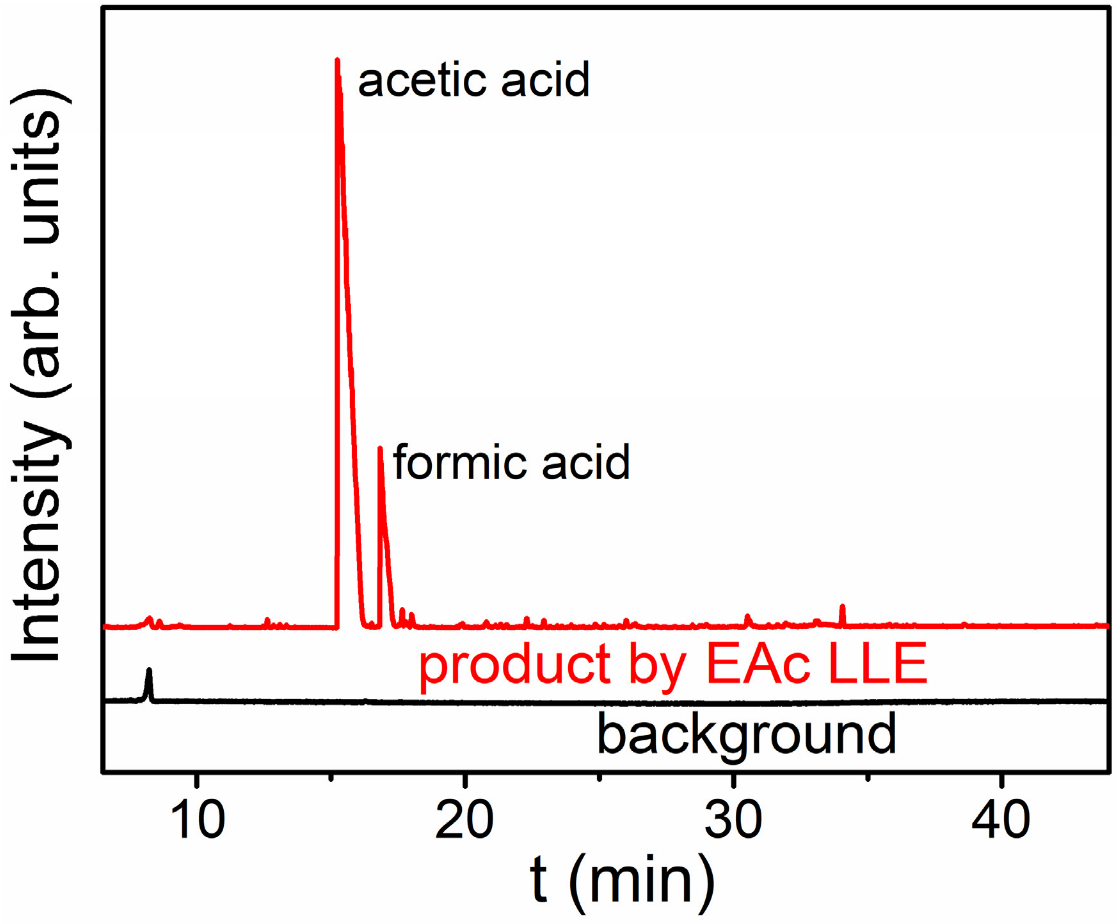

Supplementary Figure 30| Identification of organic acid product from M-COR. GC-MS analysis of anodic reaction products (red curve) after liquid-liquid extraction (LLE). The black curve is spectrum of background (solvent).

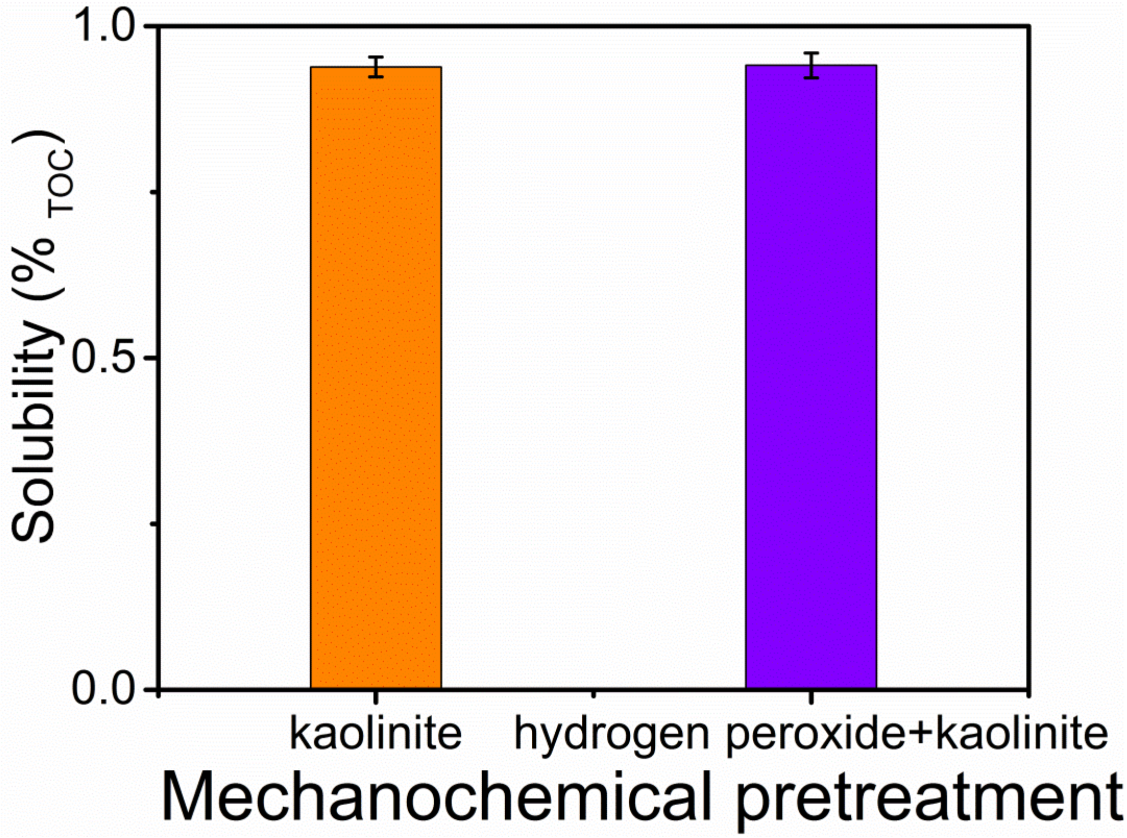

Supplementary Figure 31| Dissolution enhancement. TOC based solubility comparison with and without adding of hydrogen peroxide during the kaolinite assisted ball mill pretreatment. Error bar stands for standard deviation and more than four samples were collected each.

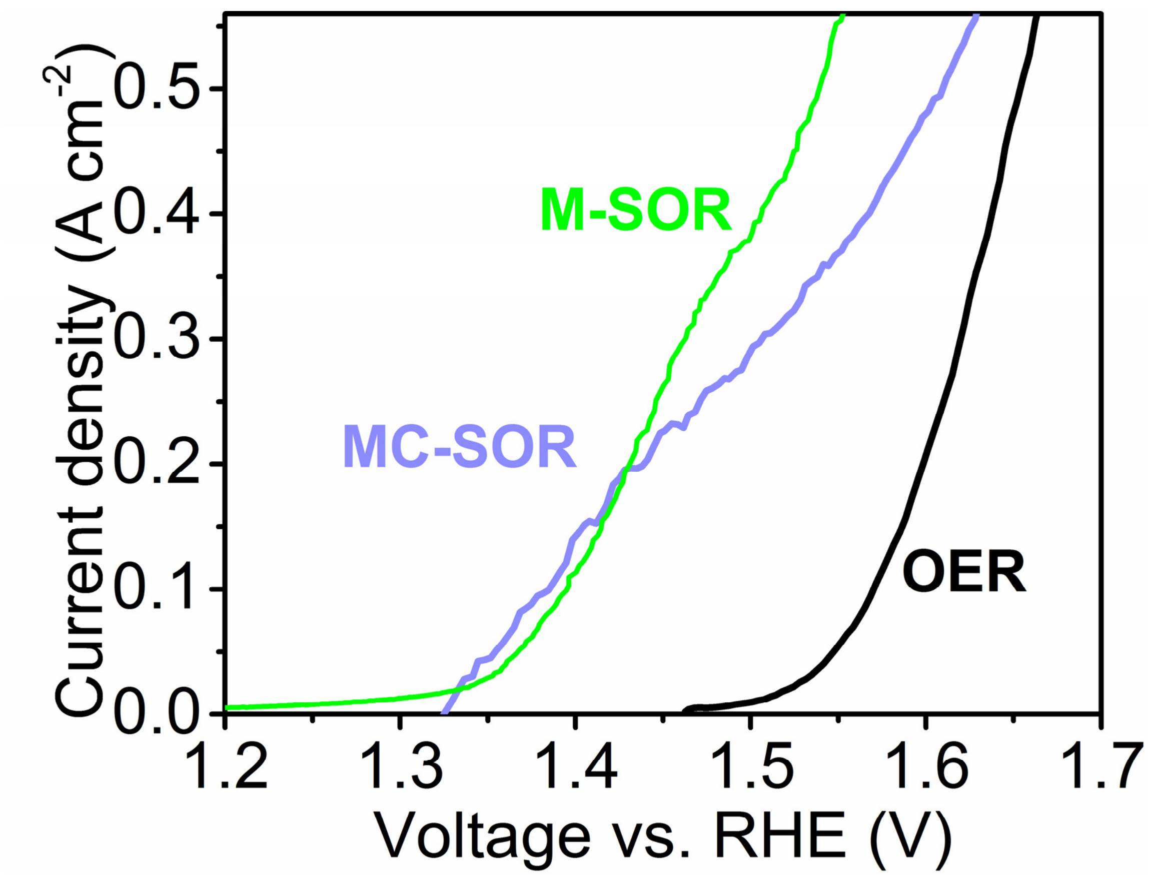

Supplementary Figure 32| Electrochemical measurement. Linear sweep voltammograms of anodic reactions at 5 mV s^{-1} in 1.0 M KOH and at the condition of containing 6.0 g L^{-1} of milled shrimp shell (M-SOR) and H_2O_2 catalyst-assisted milled shrimp shell (MC-SOR).

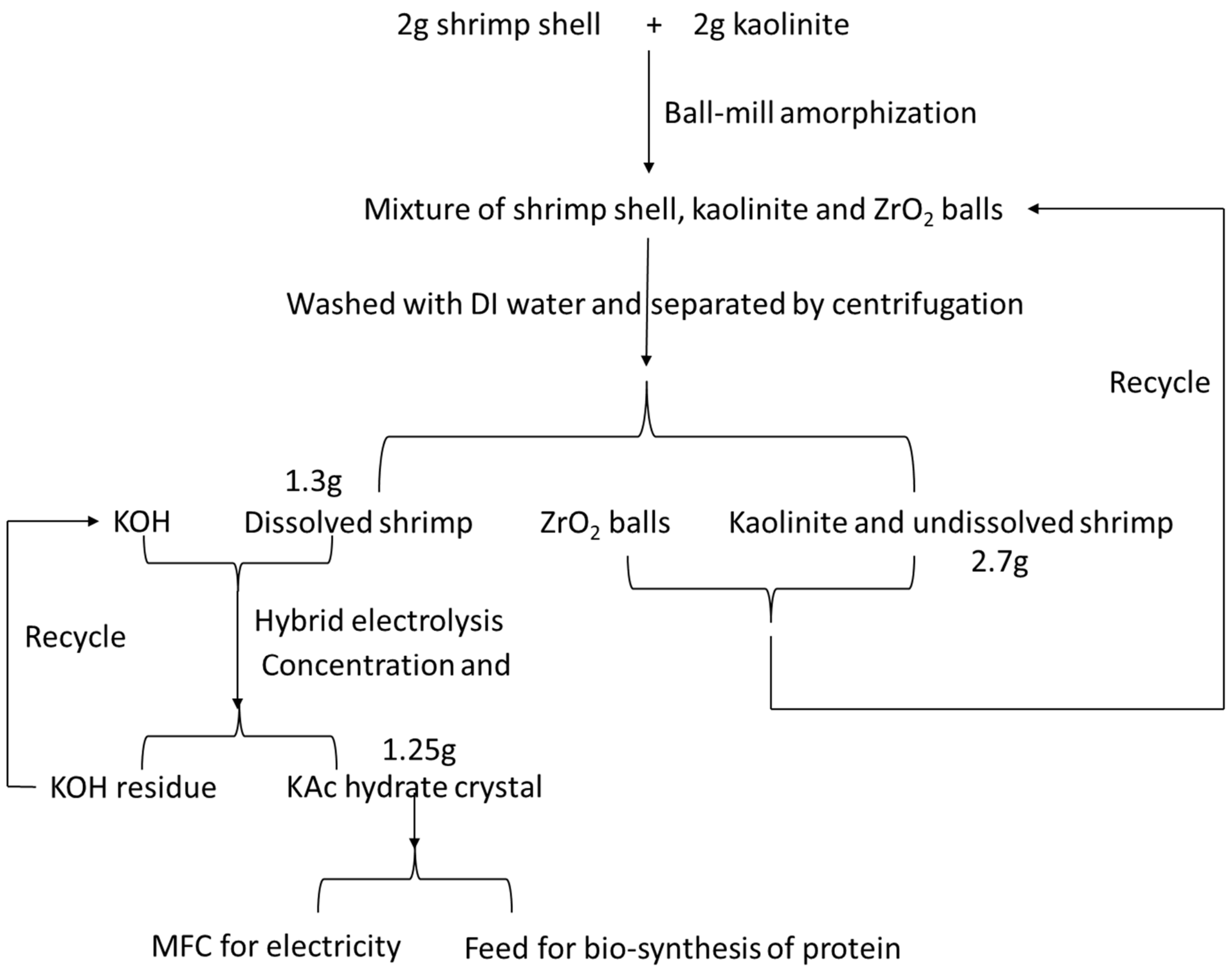

Supplementary Figure 33| Material balance. The cycling diagram of the whole process and corresponding material balance.

Supplementary Table 1| Randles circuit fitted impedance for OER, COR+OER and M-COR at open circuit potential.

	RS (OHM)	RCT (OHM)	CPE (OHM $S^{-0.5}$)
**OER**	1.42	265.8	49.7
**COR**	1.68	94.9	16.1
**M-COR**	1.66	37.2	13.7

Supplementary Table 2| Measurement condition of PV-driven electrolyzer. The electrode area is 28 cm^2. The series resistance is obtained from EIS measurement. All the current and potential values are average values under intermittent solar flux obtained from the voltage and current meters connected in the circuit. The experiment was conducted on the roof of our laboratory (N 1º20'54.4"; E 103º40'59.3") at 2-5 pm on 14 June 2019 (partially cloudy).

		**Pure water splitting**	**Hybrid electrolysis**
**Strong solar irradiation**	**current (A)**	~14	~15
	**current density ($mA\ cm^{-2}$)**	~500	~535
	**potential (V)**	~3.8	~3.6
**Weak solar irradiation**	**Current (A)**	~8.4	~9.5
	**current density ($mA\ cm^{-2}$)**	~300	~339
	**Potential (V)**	~3.1	~2.9
**EIS measurement**	**Rs (Ω)**	0.13	0.12

Supplementary Table 3| Comparison of our hybrid electrolysis and conventional lignocellulose electrolysis.

Anodic oxidation (Biomass)	Electrode	Current density	Product selectivity	Remarks	Ref.
Black liquor	-	<2mA cm^{-2}	No product analysis	No quantification of hydrogen rate	30
Kraft lignin	Pt, Au, Ni, Cu, DSA-O_2 and PbO_2	<3mA cm^{-2}	<17% selectivity to Vanillin	No hydrogen gathering	31
Black liquor	platinum wire	<10 $mAcm^{-2}$	No product analysis	No quantification of hydrogen	32
Cellulose	glassy carbon plates	20-40mA	No specific product	No hydrogen gathering<br>TEMPO waste	33
Regenerated cellulose fiber	glassy carbon	<10mA	No specific product	No hydrogen gathering<br>Long reaction duration (45 h)	34
Cellulose and cellobiose	Gold electrode	1-10mA	No product analysis	No hydrogen gathering	35
Cellulose	graphite/PTFE electrodes	<10mA	Soluble sugar and HMF (<15.8%)	No hydrogen gathering<br>Electro-Fenton degradation	36
Cellulose oligosaccharides	MnO_2/graphite/PTF E	<10mA	High selectivity to glucose	No hydrogen gathering<br>Similar to hydrolysis process	37
Cellulose	Gold electrode	<1mA cm^{-2}	No product analysis	No hydrogen gathering	38
Hemicellulose	Gold electrode	<1mA cm^{-2}	No product analysis	No hydrogen gathering	39
Lignin	Non-precious metal nanoparticle	<5mA	No specific product	No hydrogen gathering	40
Cellulose	AuNPs/C	<1mA	No product analysis	No hydrogen gathering	41
Cellulose	Au/carbon aerogel	-	Gluconate (67.8%)	No hydrogen gathering	42
Lignin derivatives	PtFe/C	0.1–0.2 mA cm^{-2}	90% selectivity to CO2	10-30mmolmin^{-1} H_2 production<br>temp: 100-170 °C	43
Lignocellulose mono- and disaccharide derivatives	PtFe/C	0.15–0.2 mA cm^{-2}	Near 100% selectivity to CO2	Cellulose biomass fuel cell	44
Crushed barley straw	nickel foam	< 3mA cm^{-2}	No specific product	No hydrogen gathering Solvent<br>(5M NaOH or 65 wt% $ZnCl_2$)	45
High temperature treated Food waste	carbonyl-group functionalized mesoporous carbon	0.15–0.25 mA cm^{-2}	90% selectivity to CO_2	85% H_3PO_4 solvent at a temperature of 150 °C<br>30-40 mmol min^{-1} H_2 production concomitant of CO_2	46
Lignocellulose derivatives (including $C_{14}H_{14}O_8Na$ and $C_5H_7O_3$)	Ketjen black carbon	0.15–0.25 mA cm^{-2}	90% selectivity to CO2	30-40mmol min^{-1} H_2 production<br>concomitant of CO2<br>85% H_3PO_4 170 °C	47
Lignin	Pt-Ru catalyst	<3mA cm^{-2}	No product analysis	Polymer electrolyte membrane (PEM) reactor with flow mode temp: 30-90 °C	48
Lignin and its model compounds	glassy carbon (GC)	25-80 mA	No specific product	No hydrogen gathering<br>TEMPO waste	49
Chitin and raw crustacean shell	hp-Ni	>1A cm^{-2}	Near 100% selectivity to acetic acid	Up to 5L h^{-1} hydrogen production rate<br>Single compartment cell<br>No separation issues (high selectivity to HAc)<br>Driven by intermittent solar energy<br>Acetic acid can be further cycled to produce protein<br>No waste produced & sustainable	This work

Supplementary Table 4| Comparison of key features between our hybrid electrolysis and AWE.

Parameters \ Electrolysis	AWE	Hybrid electrolysis
Reactants	Pure water	Water and raw biomass
Pretreatment of reactants	Purify, heating	Mechanochemical treatment of raw biomass
Products	Hydrogen and oxygen	Hydrogen and acetate (nitrate)
Value of H_2	>99.9% with pressure up to 30 bar	Potentially > 99 % pressurized H_2
membrane	Diaphragm membrane	Potentially membraneless
Operating potential	1.8-2.4 @ 0.2-0.4 A/cm^2	~10% less voltage needed
Energy efficiency	70-80%	80-90% excluding pretreatment energy
Operating temperature	60-80 ^{o}C	Room temperature
Operation condition	Restart at 2% H_2 crossover	Potentially continuous operation

Supplementary Table 5| Estimation of the cost of anodic reactants and products of our hybrid electrolysis.

Property / Material	Price ($/kg)	Total mass for 1 kg H_2 produced (kg)	Total value ($)	Role in the electrolysis
Dry shrimp shell for chitin extraction	0.1	78.9	8	Anodic reactants
Potassium hydroxide	0.8	35.1	28	Electrolyte
Potassium acetate	1.0	49.1	49	Anodic products

*1. based on $C_8H_{15}NO_6 + 5OH^- \rightarrow 4C_2H_3O_2^- + 4H_2 + NO_3^-$
2. every 1g dry shrimp shell contains 0.35g chitin
3. for 1 kg H_2 produced

Supplementary Table 6| Energy consumption for hybrid electrolysis compared to that of the state-of-the-art AWE.

Parameters \ Electrolysis	AWE	Hybrid electrolysis
Pretreatment step	Water purification, heating	Ball mill of raw biomass
Operating potential	1.8 V @ 0.2 A/cm^2	~0.17 V less voltage needed @ 0.2 A/cm^2
Efficiency	70%	~80%
Operating temperature	Maintain at 60-80 ^{o}C	Room temperature
Operation condition	System restart at 2% H_2 crossover	Potentially continuous operation

Supplementary Table 7| Comparison of electricity-to-hydrogen efficiency.

Electrolysis / Parameters	The state-of-the-art AWE	Hybrid electrolysis with grid electricity
Pretreatment step	Water purification and heating	Ball mill (950 MJ/317 MJ)
Electricity consumed	198 MJ	178 MJ
Energy of H_2 generated	142 MJ	142 MJ
Electricity-to-hydrogen efficiency	72 %	80% (12.5 %/28.7%)

*1. Assume the energy consumption in pump, H_2 purification, system restarting etc. are the same for all systems.
2. Based on 1 kg H_2 produced, which needs 27.6 kg chitin.

Supplementary References:

1. Margoutidis G, Parsons VH, Bottaro CS, Yan N, Kerton FM. Mechanochemical Amorphization of α-Chitin and Conversion into Oligomers of N-Acetyl-d-glucosamine. *ACS Sustain. Chem. Eng.* **6**, 1662-1669 (2018).
2. Ripatti DS, Veltman TR, Kanan MW. Carbon Monoxide Gas Diffusion Electrolysis that Produces Concentrated C2 Products with High Single-Pass Conversion. *Joule* **3**, 240-256 (2019).
3. Hulsen T, Batstone DJ, Keller J. Phototrophic bacteria for nutrient recovery from domestic wastewater. *Water. Res.* **50**, 18-26 (2014).
4. Hülsen T, Hsieh K, Lu Y, Tait S, Batstone DJ. Simultaneous treatment and single cell protein production from agri-industrial wastewaters using purple phototrophic bacteria or microalgae – A comparison. *Bioresour. Technol.* **254**, 214-223 (2018).
5. Kasaai MR. Determination of the degree of N-acetylation for chitin and chitosan by various NMR spectroscopy techniques: A review. *Carbohydr. Polym.* **79**, 801-810 (2010).
6. Chen X, Yang H, Zhong Z, Yan N. Base-catalysed, one-step mechanochemical conversion of chitin and shrimp shells into low molecular weight chitosan. *Green Chem.* **19**, 2783-2792 (2017).
7. Marangoni DG, Wylie IG, Roscoe SG. Surface electrochemistry of the oxidation reactions of α-and β-alanine at a platinum electrode. *J. Electroanal. Chem.* **320**, 269-284 (1991).
8. Vijh AK, Conway BE. Electrode Kinetic Aspects of the Kolbe Reaction. *Chem. Rev.* **67**, 623-664 (1967).
9. Zhong G*, et al.* Catalytic Enantioselective Retro‐Aldol Reactions: Kinetic Resolution of β‐Hydroxyketones with Aldolase Antibodies. *Angew. Chem. Int. Ed.* **37**, 2481-2484 (1998).
10. Vinu R, Broadbelt LJ. A mechanistic model of fast pyrolysis of glucose-based carbohydrates to predict bio-oil composition. *Energy Environ. Sci.* **5**, (2012).
11. Degenstein JC*, et al.* Fast pyrolysis of 13C-labeled cellobioses: gaining insights into the mechanisms of fast pyrolysis of carbohydrates. *J. Org. Chem.* **80**, 1909-1914 (2015).
12. Luo S, Xu H, Zhang L, Li J, Cheng J-P. Highly Enantioselective Direct syn- and anti-Aldol Reactions of Dihydroxyacetones Catalyzed by Chiral Primary Amine Catalysts. *Org. Lett.* **10**, 653-656 (2008).
13. Huyghues-Despointes A, Yaylayan VA. Retro-Aldol and Redox Reactions of Amadori Compounds: Mechanistic Studies with Variously Labeled d-[13C]Glucose. *J. Agric. Food Chem.* **44**, 672-681 (1996).
14. Yamagaki T, Suzuki H, Tachibana K. Semiquantitative analysis of isomeric oligosaccharides by negative-ion mode UV-MALDI TOF postsource decay mass spectrometry and their fragmentation mechanism study atN-acetyl hexosamine moiety. *J. Mass Spectrom.* **41**, 454-462 (2006).
15. So S, Wille U, da Silva G. A Theoretical Study of the Photoisomerization of Glycolaldehyde and Subsequent OH Radical-Initiated Oxidation of 1,2-Ethenediol. *J. Phys. Chem. A* **119**, 9812-9820 (2015).
16. Mardyukov A, Eckhardt AK, Schreiner PR. 1,1-Ethenediol: The Long Elusive Enol of Acetic Acid. *Angew. Chem. Int. Ed.* **59**, 5577-5580 (2020).
17. Degenstein JC*, et al.* Fast pyrolysis of 13C-labeled cellobioses: Gaining insights into the mechanisms of fast pyrolysis of carbohydrates. *J. Org. Chem.* **80**, 1909-1914 (2015).

18. Wakatsuki K. Acetyls Chain-World Market Overview. In: *Asia Petrochemical Industry Conference* (2015).
19. Zhen G, Lu X, Kato H, Zhao Y, Li Y-Y. Overview of pretreatment strategies for enhancing sewage sludge disintegration and subsequent anaerobic digestion: Current advances, full-scale application and future perspectives. *Renew. Sust. Energ. Rev.* **69**, 559-577 (2017).
20. Wang Y*, et al.* Self-Assembly-Induced Mosslike Fe_2O_3 and FeP on Electro-oxidized Carbon Paper for Low-Voltage-Driven Hydrogen Production Plus Hydrazine Degradation. *ACS Sustain. Chem. Eng.* **6**, 15727-15736 (2018).
21. Merzendorfer H, Zimoch L. Chitin metabolism in insects: structure, function and regulation of chitin synthases and chitinases. *J. Exp. Biol.* **206**, 4393-4412 (2003).
22. Hoell IA, Vaaje-Kolstad G, Eijsink VGH. Structure and function of enzymes acting on chitin and chitosan. *Biotechnol. Genet. Eng. Rev.* **27**, 331-366 (2010).
23. Schalenbach M, Zeradjanin AR, Kasian O, Cherevko S, Mayrhofer KJJ. A Perspective on Low-Temperature Water Electrolysis – Challenges in Alkaline and Acidic Technology. *Int. J. Electrochem. Sci.* **13**, 1173-1226 (2018).
24. Ursua A, Gandia LM, Sanchis P. Hydrogen Production From Water Electrolysis: Current Status and Future Trends. *Proc. IEEE* **100**, 410-426 (2012).
25. Carmo M, Fritz DL, Mergel J, Stolten D. A comprehensive review on PEM water electrolysis. *Int. J. Hydrog. Energy* **38**, 4901-4934 (2013).
26. Kaufman Rechulski MD, Käldström M, Richter U, Schüth F, Rinaldi R. Mechanocatalytic Depolymerization of Lignocellulose Performed on Hectogram and Kilogram Scales. *Ind. Eng. Chem. Res.* **54**, 4581-4592 (2015).
27. Chang KLB, Tai M-C, Cheng F-H. Kinetics and Products of the Degradation of Chitosan by Hydrogen Peroxide. *J. Agric. Food Chem.* **49**, 4845-4851 (2001).
28. Thorwirth R, Bernhardt F, Stolle A, Ondruschka B, Asghari J. Switchable selectivity during oxidation of anilines in a ball mill. *Chem. Eur. J.* **16**, 13236-13242 (2010).
29. Yao SG*, et al.* Mechanochemical Treatment Facilitates Two-Step Oxidative Depolymerization of Kraft Lignin. *ACS Sustain. Chem. Eng.* **6**, 5990-5998 (2018).
30. Roy Ghatak H. Electrolysis of black liquor for hydrogen production: Some initial findings. *Int. J. Hydrog. Energy* **31**, 934-938 (2006).
31. Parpot P, Bettencourt AP, Carvalho AM, Belgsir EM. Biomass conversion: attempted electrooxidation of lignin for vanillin production. *J. Appl. Electrochem.* **30**, 727-731 (2000).
32. Ghatak HR, Kumar S, Kundu PP. Electrode processes in black liquor electrolysis and their significance for hydrogen production. *Int. J. Hydrog. Energy* **33**, 2904-2911 (2008).
33. Parpot P, Servat K, Bettencourt AP, Huser H, Kokoh KB. TEMPO mediated oxidation of carbohydrates using electrochemical methods. *Cellulose* **17**, 815-824 (2010).
34. Isogai T, Saito T, Isogai A. TEMPO Electromediated Oxidation of Some Polysaccharides Including Regenerated Cellulose Fiber. *Biomacromolecules* **11**, 1593-1599 (2010).
35. Sugano Y, Vestergaard Md, Yoshikawa H, Saito M, Tamiya E. Direct Electrochemical Oxidation of Cellulose: A Cellulose-Based Fuel Cell System. *Electroanalysis* **22**, 1688-1694 (2010).
36. Wang Z-X, Li G, Yang F, Chen Y-L, Gao P. Electro-Fenton degradation of cellulose using graphite/PTFE electrodes modified by 2-ethylanthraquinone. *Carbohydr. Polym.* **86**, 1807-1813 (2011).
37. Yang F, Zhang Q, Fan H-X, Li Y, Li G. Electrochemical control of the conversion of cellulose oligosaccharides into glucose. *J. Ind. Eng. Chem.* **20**, 3487-3492 (2014).

38. Sugano Y, Latonen R-M, Akieh-Pirkanniemi M, Bobacka J, Ivaska A. Electrocatalytic Oxidation of Cellulose at a Gold Electrode. *ChemSusChem* **7**, 2240-2247 (2014).
39. Sugano Y, Saloranta T, Bobacka J, Ivaska A. Electro-catalytic oxidation of hemicelluloses at the Au electrode. *Phys. Chem. Chem. Phys.* **17**, 11609-11614 (2015).
40. Movil O, Garlock M, Staser JA. Non-precious metal nanoparticle electrocatalysts for electrochemical modification of lignin for low-energy and cost-effective production of hydrogen. *Int. J. Hydrog. Energy* **40**, 4519-4530 (2015).
41. Sugano Y*, et al.* Specific Electrocatalytic Oxidation of Cellulose at Carbon Electrodes Modified by Gold Nanoparticles. *ChemCatChem* **8**, 2401-2405 (2016).
42. Xiao H, Wu M, Zhao G. Electrocatalytic Oxidation of Cellulose to Gluconate on Carbon Aerogel Supported Gold Nanoparticles Anode in Alkaline Medium. *Catalysts* **6**, 5 (2016).
43. Hibino T, Kobayashi K, Nagao M, Teranishi S. Hydrogen Production by Direct Lignin Electrolysis at Intermediate Temperatures. *ChemElectroChem* **4**, 3032-3036 (2017).
44. Hibino T, Kobayashi K, Lv P, Nagao M, Teranishi S. High Performance Anode for Direct Cellulosic Biomass Fuel Cells Operating at Intermediate Temperatures. *Bull. Chem. Soc. Jpn.* **90**, 1017-1026 (2017).
45. Li S, Song X. Study on the preparation and production factors of a direct lignocellulose biomass fuel cell. *J. Electroanal. Chem.* **810**, 55-61 (2018).
46. Hibino T*, et al.* Efficient Hydrogen Production by Direct Electrolysis of Waste Biomass at Intermediate Temperatures. *ACS Sustain. Chem. Eng.* **6**, 9360-9368 (2018).
47. Hibino T, Kobayashi K, Ito M, Nagao M, Fukui M, Teranishi S. Direct electrolysis of waste newspaper for sustainable hydrogen production: an oxygen-functionalized porous carbon anode. *Appl. Catal. B-Environ.* **231**, 191-199 (2018).
48. Caravaca A, Garcia-Lorefice WE, Gil S, de Lucas-Consuegra A, Vernoux P. Towards a sustainable technology for H_2 production: Direct lignin electrolysis in a continuous-flow Polymer Electrolyte Membrane reactor. *Electrochem. Commun.* **100**, 43-47 (2019).
49. Rafiee M, Alherech M, Karlen SD, Stahl SS. Electrochemical Aminoxyl-Mediated Oxidation of Primary Alcohols in Lignin to Carboxylic Acids: Polymer Modification and Depolymerization. *J. Am. Chem. Soc.* **141**, 15266-15276 (2019).
