# Supplementary material for: Raw biomass electroreforming coupled to green hydrogen generation
Source: Nat Commun. 2021 Mar 31;12:2008. doi: 10.1038/s41467-021-22250-9 (PMC8012647; doi:10.1038/s41467-021-22250-9)
Supplement: Supplementary file 2 — Description of Additional Supplementary Files [file 41467_2021_22250_MOESM2_ESM.pdf]

Description of Additional Supplementary Files

File Name: Supplementary Movie 1
Description: Solar-driven single-compartment hybrid electrolysis for simultaneous hydrogen production and chitin electroreforming.
